# Ligand-based and structure-based studies to develop predictive models for SARS-CoV-2 main protease inhibitors through the 3d-qsar.com portal

**DOI:** 10.1007/s10822-022-00460-7

**Published:** 2022-06-18

**Authors:** Eleonora Proia, Alessio Ragno, Lorenzo Antonini, Manuela Sabatino, Milan Mladenovič, Roberto Capobianco, Rino Ragno

**Affiliations:** 1 Department of Drug Chemistry and Technology, Rome Center for Molecular Design, Sapienza University of Rome, P.le Aldo Moro 5, 00185 Rome, Italy; 2 Department of Computer, Control and Management Engineering “Antonio Ruberti”, Sapienza University of Rome, Rome, Italy; 3 Department of Chemistry, Faculty of Science, Kragujevac Center for Computational Biochemistry, University of Kragujevac, Radoja Domanovića 12, P.O. Box 60, 34000 Kragujevac, Serbia; 4 Sony AI, Rome, Italy

**Keywords:** 3-D QSAR, COMBINE, SARS-Cov-2, Ligand-based drug design, Structure-based drug design, Structure-activity relationships

## Abstract

The main protease (M^pro^) of SARS-Cov-2 is the essential enzyme for maturation of functional proteins implicated in viral replication and transcription. The peculiarity of its specific cleavage site joint with its high degree of conservation among all coronaviruses promote it as an attractive target to develop broad-spectrum inhibitors, with high selectivity and tolerable safety profile. Herein is reported a combination of three-dimensional quantitative structure–activity relationships (3-D QSAR) and comparative molecular binding energy (COMBINE) analysis to build robust and predictive ligand-based and structure-based statistical models, respectively. Models were trained on experimental binding poses of co-crystallized M^pro^-inhibitors and validated on available literature data. By means of deep optimization both models’ goodness and robustness reached final statistical values of *r*^*2*^/*q*^*2*^ values of 0.97/0.79 and 0.93/0.79 for the 3-D QSAR and COMBINE approaches respectively, and an overall predictiveness values of 0.68 and 0.57 for the SDEP_PRED_ and AAEP metrics after application to a test set of 60 compounds covered by the training set applicability domain. Despite the different nature (ligand-based and structure-based) of the employed methods, their outcome fully converged. Furthermore, joint ligand- and structure-based structure–activity relationships were found in good agreement with nirmatrelvir chemical features properties, a novel oral M^pro^-inhibitor that has recently received U.S. FDA emergency use authorization (EUA) for the oral treatment of mild-to-moderate COVID-19 infected patients. The obtained results will guide future rational design and/or virtual screening campaigns with the aim of discovering new potential anti-coronavirus lead candidates, minimizing both time and financial resources. Moreover, as most of calculation were performed through the well-established web portal *3d-qsar.com* the results confirm the portal as a useful tool for drug design.

**Graphical abstract:**

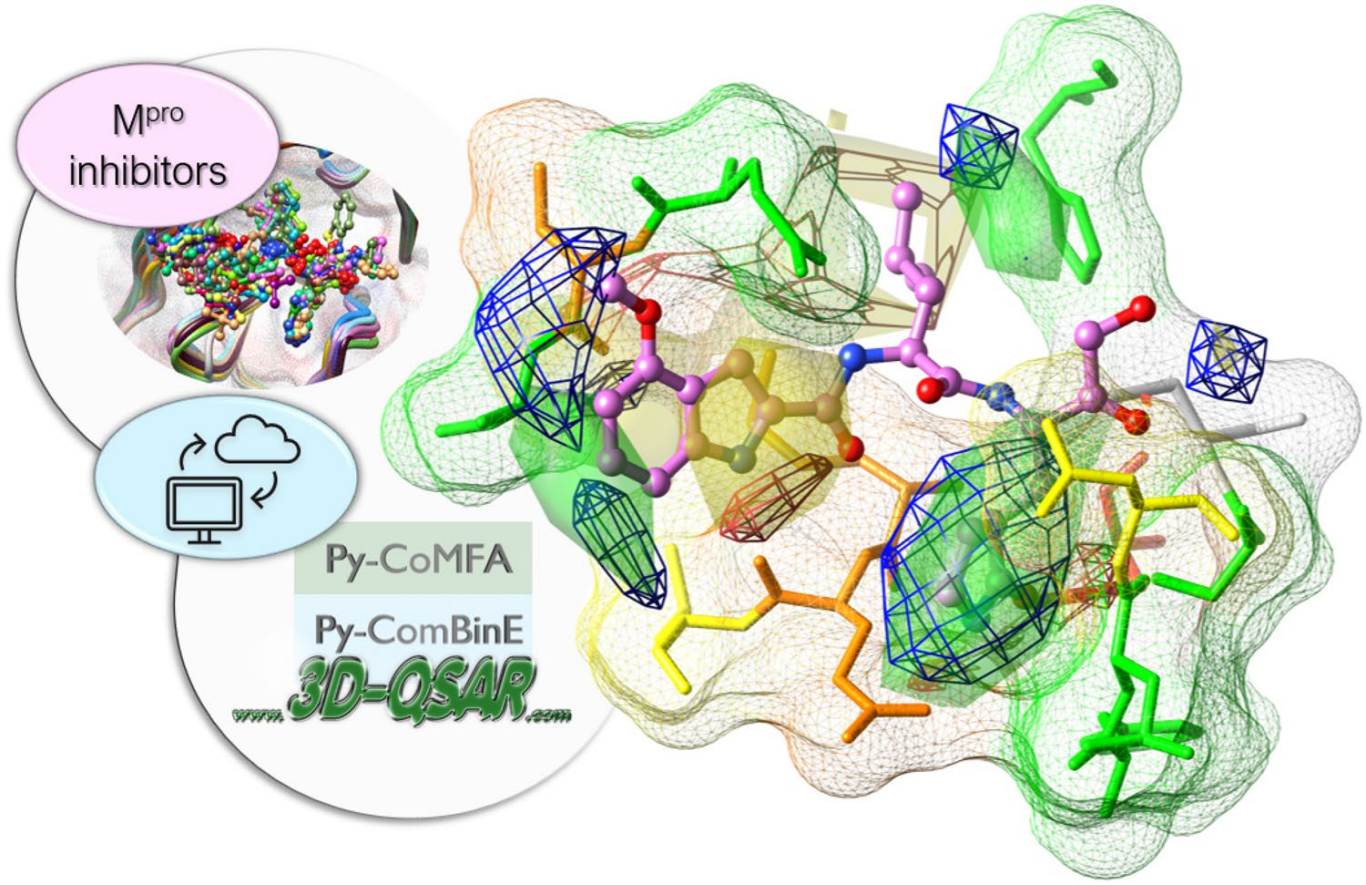

**Supplementary Information:**

The online version contains supplementary material available at 10.1007/s10822-022-00460-7.

## Introduction

In December 2019, a previously unknown human coronavirus was reported to be the etiological agent of a pneumonia that occurred in a cluster of patients in Wuhan, capital of the Hubei province in China; in a few months this coronavirus rapidly spread throughout the world [1, 2]. The World Health Organization (WHO) named the infectious disease coronavirus disease (COVID-19) [3] and declared the outbreak a global pandemic on 11 March 2020 as the first pandemic caused by a coronavirus [4]. Coronaviruses (CoVs) are a large group of enveloped, positive-sense, single-stranded RNA viruses that cause a wide variety of diseases in humans and other animals. The International Committee on Taxonomy of Viruses designated the responsible etiological agent of COVID-19 as *severe acute respiratory syndrome coronavirus 2* (SARS-CoV-2). It taxonomically clusters with SARS-related CoVs, within the *Betacoronavirus* genus, *Coronaviridiae* family [5].

At present, seven coronaviruses are known to cause human diseases [6], the low-pathogenicity members (HCoV-NL63, HCoV-229E, HCoV-OC43 and HKU1) generally lead to mild to moderate upper respiratory illness, such as common cold or pneumonia, whereas the highly pathogenic members (SARS-CoV, MERS-CoV, SARS-CoV-2) are known to cause severe respiratory diseases with high morbidity and lethality. Outbreaks of new human highly pathogenic coronavirus infections have periodically emerged from animal reservoirs, including severe acute respiratory syndrome (SARS) in 2003 [7] and Middle East respiratory syndrome (MERS) in 2012 [8]: SARS-CoV-2 marks the third introduction of a highly pathogenic CoV into the human population within the last two decades. Furthermore, it is readily transmitted from human to human and it has spread at an alarming speed, posing a significant threat to public global health.

Genetic sequence analysis revealed that SARS-CoV-2 shares respectively 79.6% and about 50% of genome sequence identity with the other zoonotic SARS-CoV and MERS-CoV [9]; exceptionally comparison with bat coronavirus, SL-CoV-RaTG13, showed a whole-genome sequence identity of 96.2% [10]. This phylogenetic relationship provided evidence SARS-CoV-2 may have originated from bats and emerged in humans by an intermediate host, similarly to both SARS and MERS outbreaks [1, 11]. SARS-CoV-2 genome contains at least six open reading frames (ORFs) [10]; the first two overlapping ORF 1a/b at the 5′ end terminal, encode for polyproteins pp1a and pp1ab. The well-characterized main protease (M^pro^), also known as the 3C-like protease (3CL^pro^), cleaves an extensive part of the precursor polyproteins into individual and functional proteins, which form the replicase/transcriptase complex (RTC). M^pro^ is a three-domain (domains I to III) cysteine protease, and its active form is a dimer where each protomer features a noncanonical Cys145-His41 catalytic dyad located in a wide cleft between domain I and II. It operates at no less than 11 conserved cleavage sites that share the Leu-Gln↓Ser(Ala, Gly) (↓ indicates the cleavage site) as preferred recognition sequence, including its own autolytic cleavage from pp1a. Moreover, M^pro^ has a unique substrate preference for glutamine in P1 site, an absent feature in closely related host proteases, suggesting it is feasible to achieve high selectivity and acceptable safety profile on this target [12, 13]. M^pro^ has a pivotal role in the life cycle of CoVs: its highly conserved catalytic domain among all CoVs promotes it as an attractive drug target for broad-spectrum anti-coronavirus therapy. In order to discover effective drugs against the novel coronavirus, two main approaches have been pursued: drug repurposing of already existing drugs and rational design of new selective compounds [13, 14]. The repurposing approach allows for rapid identification of potential drug leads through massive screening of libraries of approved and investigational drugs, often automated by means of fragment screenings and high throughput screening (HTS). It allowed to quickly start clinical trials with safe-in-man compounds that exhibits only modest experimental antiviral evidence. Through this route, several inhibitors have been reported from HTS [13, 15], libraries of proteases [16] and bioactive components of traditional Chinese medicine [17–19]. In addition, repurposing offers the advantage to exploit the considerable amount of data reported on other human pathogenic CoVs over the past decade and hopefully accelerate drug discovery process [13, 20–26].

However, the scientific community agrees that the most favored strategy to obtain safe and efficacious drugs is the coherent design of *ad-hoc* chemical entities. Such an approach requires the knowledge of the target and the substrates. Several covalent reversible inhibitors, that efficiently compete with the natural substrates, have already been reported. The compounds included α-ketoamide analogs [22, 27, 28], peptidomimetic aldehydes [21, 25, 29–31] and various ketones derivatives [20, 32].

**Fig. 1:**
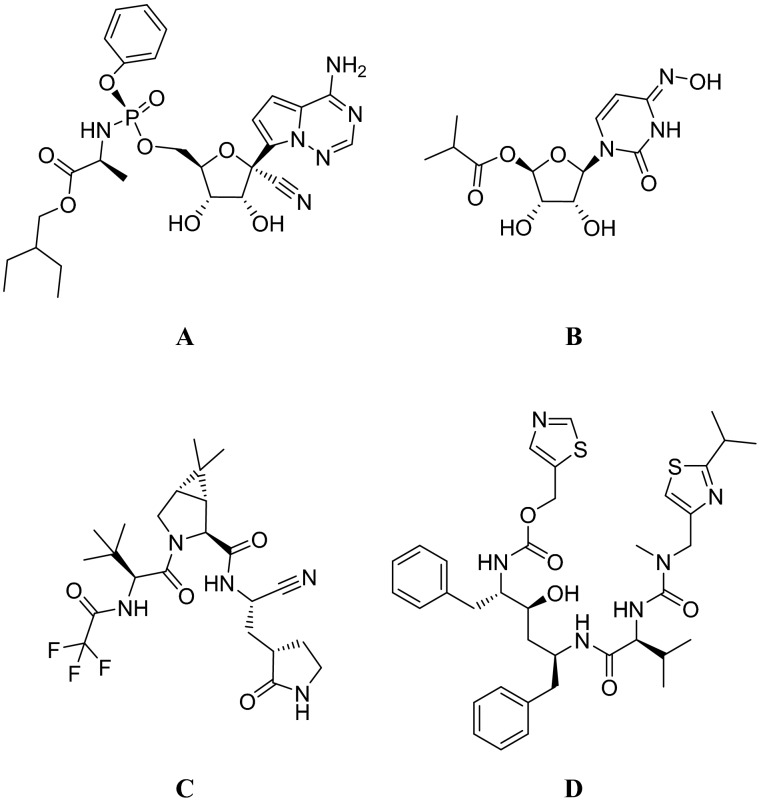
Currently FDA-approved or authorized antivirals used for the treatment of mild-to-moderate COVID-19: remdesivir (**A**), molnupiravir (**B**), nirmatrelvir (**C**) and ritonavir (**D**)

Notwithstanding the accelerated COVID-19 vaccines [33] development pipeline, the recurrent emergence of new coronaviruses able to jeopardize public health highlights the urgent need for developing effective drugs against pathogenic coronaviruses. In fact, while preparing this report, only three drugs have been authorized by the U.S. Food and Drug Administration (FDA) for the treatment of COVID-19 in patients at high risk for progression to severe disease. Veklury (remdesivir) (Fig. 1, panel A) and Lagevrio (molnupiravir) (Fig. 1, panel B) are nucleoside analogues targeting the viral RNA-dependent RNA polymerase (RdRp). Remdevisivir, despite some initial conflicting opinions and contrasting trials [34–36], has been recently approved [37] for intravenous (IV) use, while molnupiravir received an emergency use authorization (EUA) [38] for oral use. More recently, FDA issued an EUA [39] for Paxlovid (nirmatrelvir co-packaged with ritonavir, Fig. 1, panels C and D) for oral use, a combination of a pan-coronaviruses M^pro^ inhibitor [40] with an HIV protease inhibitor exerting inhibitory activity against CYP3A4 boosting nirmatrelvir serum levels.

Due to the urgent need to develop COVID-19 drugs, computational methods to rationally design new anti-coronavirus agents have been largely applied also in combination with crystallographic experimental data, but few of them exploited any combination of ligand-based (LB) and structure-based (SB) approaches.

Herein, are reported LB and SB computational approaches applied to a series of SARS-CoV-2 M^pro^ inhibitors. Through the Py-CoMFA and Py-ComBinE applications available on the 3d-qsar.com portal [41], robust and predictive comparative molecular field analysis (CoMFA) [42] and comparative molecular binding energy analysis (COMBINE) [43–47] as LB and SB three-dimensional quantitative structure–activity relationships (3-D QSAR) [48] models were built to shed light on structural molecular determinant and inhibitor/protein residues interactions mainly responsible for the M^pro^ inhibitory potency. As most of the calculations were run on 3d-qsar.com portal [41] this report demonstrates the potentiality of the available web apps as tools to develop predictive models.

## Computational methods

### Dataset preparation

The web portal 3d-qsar.com [41] freely allows to build CoMFA and COMBINE models readily handling the classical steps that need to be accomplished up to the final optimization and validation of robust models. The flowchart consists of a careful selection and alignment of training and test set molecules, calculation of molecular interaction fields (MIFs), statistical analysis, interpretation of results and graphical plots. Once these steps have been assessed, the model can be applied to predict the activity of untested molecules or to design new chemical entities with either LB or SB approaches.

#### Training set and test set compilation

Despite the huge amount of data generated for COVID-19 at the time this investigation was started, the lack of homogeneity of condition assays, protocols and experimental bioactivities units of measurement prevented the developing of wide and computationally applicable medicinal chemistry models. Therefore, the dataset assembling was focused on small molecule compounds with associated biological activity data as much as possible consistent with each other. For this purpose, only data of M^pro^ inhibitors whose activity was mainly expressed in terms of IC_50_ were collected from literature [13, 15–29, 31, 32] (Supporting Information Table SI1 and Table SI2). Among them, 21 were available as experimentally co-crystallized in ligand–protein complexes from the Protein Data Bank (PDB) [49] and were therefore selected to compile a training set (TR, Table 1, Supporting Information Table SI1). All TR compounds were reported as covalent inhibitors characterized by either a peptide or a peptidomimetic scaffold. Non-complexed inhibitors were selected (see dataset compilation section in Supporting Information) to compile a modeled test set (TS_MOD_, Supporting Information Table SI2) that comprised 67 molecules with different molecular scaffolds (peptidomimetic, isatin, flavonoid and others) and different putative mechanisms of action (covalent/non-covalent). Moreover, during modeling, a crystal test set (TS_CRY_, Supporting Information Table SI3) was compiled with 47 recently published [50–57] co-crystallized complexes, selected in order to maintain consistency with the bioactivity assay and measurement (IC_50_). The final TR and TS_MOD_, constituted of 21 (**1–21**) and 67 (**22**–**88**) molecules, were respectively used to build and validate 3-D QSAR and COMBINE models, while the TS_CRY_ 47 complexes (**89–135**) were used to assess models predictiveness with experimental data.

**Table 1:** The 21 Mpro inhibitors 2-D structures included in the training set and associated IC_50_ and PDB entry codes

Mol ID^a^	PDB ID^b^	Structure	IC_50_ (μM)
1	6XA4		0.97 [16]
2	6WTT		0.03 ± 0.008 [16]
			0.62 ± 0.08 [21]
			0.15 ± 0.03 [23]
3	6XHM		0.01 [20]
4	6XMK		0.48 [21]
5	6XBG		0.05 [28]
6	6XBI		0.45 [28]
7	7JPZ		0.10 [25]
8	7JQ0		0.09 [25]
9	7JQ1		0.02 [25]
10	7JQ2		0.03 [25]
11	7JQ3		0.06 [25]
12	7JQ4		0.05 [25]
13	7JQ5		0.11 [25]
14	6Y2F		0.67 [27]
15	6LZE		0.05 [29]
16	6M0K		0.04 [29]
17	7JKV		0.02 [32]
18	7BRP		3.1 ± 0.4 [22]
			4.13 ± 0.61 [16]
			8.0 ± 1.5 [23]
19	7D1O		5.1 ± 0.9 [22]
			5.73 ± 0.67 [16]
20	7K6E		18.00 [22]
21	6XCH		92.00 [22]

^a^Molecule number used in the manuscript

^b^PDB code associated to the ligand

#### Training set preparation

The 21 M^pro^-inhibitor associated complexes (Supporting Information Table SI1) were retrieved from the PDB and SB superimposed by means of PyMOL [58], using 6LZE as arbitrarily selected reference complex. The complexes were subjected to a cleaning procedure including removal of water molecules, ions and crystallization co-solutes and saved separated into ligand (key) and protein (lock). As all TR inhibitors were covalently bonded to Cys145, similarly as previously reported [59, 60] they were converted to the corresponding pre-covalent complexes by rebuilding the non-reacted species by means of Chimera Build Structure plugin. Reconstituted inhibitors were merged in the corresponding proteins and the resulting complexes were energy minimized to relax steric clashes. Residue protonation states were determined with PropKa [61] at a pH of 7.4. For the minimization, ligands’ parameters were calculated with Antechamber [62] using the last version of the general amber force field (GAFF2) [63] by means of the AM1-BCC method [64], while the ff14SB force field [65] was used for the proteins. The complexes were solvated using the four-point optimum point charge (OPC) water model [66] in an orthorhombic box adding Na^+^ or Cl^−^ ions to neutrality and setting to 12 Å the box boundaries distance from the protein using the tLeaP program included in Ambertools suite (version 18) [67]. The prepared topology and parameter files were used to run a 500 gradient descent minimization steps through the OpenMM [68] python library. As in agreement with the original COMBINE protocol [43], the Py-ComBinE web app requires an equal number of residue number for each protein, therefore all extra residues were removed by means of UCSF Chimera [69] from longer sequence proteins to match the shortest one (6XMK). The minimized and adjusted complexes, separated into keys and locks were uploaded to the web portal 3d-qsar.com through the Py-MolEdit web app to generate Py-CoMFA and Py-ComBinE [41] models as LB and SB 3-D QSAR applications, respectively.

#### Modeled test set preparation

The experimental reversibly reconstituted bound 21 TR ligands conformations were used as templates in a flexible alignment procedure by means of fkcombu [70]. According to the Tanimoto similarity index (Supporting Information Table SI4), each TS_MOD_ molecule was superimposed on the most similar reference molecule listed in the training set and merged with the associated protein (Supporting Information Table SI4). The resulting modeled TS_MOD_ complexes were geometry optimized with the same procedure described for the TR preparation. The TS_MOD_ minimized modeled complexes, separated into keys (the ligands) and locks (the proteins), were then uploaded to the web portal 3d-qsar.com in the same dataset containing the TR and marking them as test set molecules/complexes to evaluate the predictive ability of the under developing Py-CoMFA and Py-ComBinE models. SB alignment through molecular docking was also investigated using either Smina [71] or Plants [72] programs with all the available scoring functions. A preliminary docking assessment protocol proved any of the program/scoring function pair not suitable as the TR experimental poses were not reproduced with acceptable RMSD errors (Supporting Information Table SI6-SI9).

#### Crystal test set preparation

TS_CRY_ complexes were treated analogously to TR complexes and then uploaded to the web portal 3d-qsar.com to assess the developed Py-CoMFA and Py-ComBinE models.

### Py-CoMFA and Py-ComBinE models generation

By means of the above described TR, TS_MOD_ and TS_CRY_, a series of partial least square (PLS) [73] regression models were generated and validated through the Py-CoMFA and Py-ComBinE web applications (3d-qsar.com).

#### Py-CoMFA

To build 3-D QSAR models, the 3d-qsar.com Py-CoMFA web app builds-up three models each run with different combination of MIFs: electrostatic (ELE), steric (STE) and both ELE and STE (BOTH).

Models’ robustness was evaluated by means of cross-validation (CV) using either leave-one-out (CV_LOO_) or leave-some-out (CV_LSO_, with 5 random groups and 100 iterations) methods. ELE and STE MIFs were calculated using the TRIPOS force field to reproduce the original CoMFA methodology [41, 74]. To check for models endowed with acceptable statistical coefficients, preliminary models were built using the default data pre-treatment settings (Supporting Information Table SI10). The models were then subjected to a variable pre-treatment optimization (VPO), as implemented in Py-CoMFA, varying all the data pre-treatment settings (probe types, grid spacing, grid extension, dielectric constant, min/max cut-off energy value and minimum sigma, Supporting Information Table SI10). As the number of settings combinations was in the range of about ten billion, random combinations were run till no substantial increment of *q*^2^ value was reached. The best model was checked for any lack of chance correlation using Y-scrambling [75] in conjunction with CV. Still within the Py-CoMFA web app, results were analyzed and visually inspected as positive and negative contour plots, derived from either steric or electrostatic fields in the shape of colored polyhedrons as in the original CoMFA. Finally, the model predictive ability was assessed with the prepared external test sets (TS_MOD_ and TS_CRY_). As the 3d-qsar.com allows to build full LB models from scratch using SMILES structures and associated bioactivities, Py-CoMFA models were also tentatively built (see Supporting Information), but low statistically endowed models were obtained (data not shown) and therefore were not further investigated.

#### Py-ComBinE

Four type of ligand/protein interactions are implemented in the Py-ComBinE app, steric (STE), electrostatic (ELE), desolvation (DRY) and hydrogen bond (HB), therefore with the key/lock pairs dataset, all the possible 15 combinations of ligand/per-residues energetic interactions were considered: STE, ELE, DRY, HB and all their possible combinations (STE + ELE, STE + DRY, STE + HB, ELE + DRY, ELE + HB, DRY + HB, STE + ELE + DRY, STE + ELE + HB, STE + DRY + HB, ELE + DRY + HB, STE + ELE + DRY + HB). Differently from the original COMBINE method, the STE, ELE, DRY and HB interaction energies were calculated by means of a the using the AutoDockTools python utilities using the AutoDock 4.2 force field [76] directly on the Mpro-inhibitor complexes [43]. The combined interactions were block scaled similarly as described by Ortiz et al. [77] The combination that led to the model endowed with the highest statistical coefficients was then optimized by means of a simulated annealing feature selection (SAFS) algorithm as implemented in the Py-ComBinE web app. During all calculations cross-validation (CV_LOO_ and CV_LSO_) and Y-scrambling were used to evaluate model’s robustness and the lack of chance correlation, respectively, while the test sets were used to evaluate the predictive ability. Py-ComBinE model analysis was carried out by means of histogram plots and graphical outputs to visually characterize the most involved protein residues in modulating biological activities.

A final analysis was graphically conducted in UCSF Chimera: M^pro^ most involved residues revealed by Py-ComBinE analysis were overlapped on Py-CoMFA contour plots for a final results’ interpretation.

### Data and software availability

All computation for the 3-D QSAR and COMBINE model generation were run on the 3d-qsar.com portal (https://www.3d-qsar.com/) freely available to anyone for not profit usage, designed and maintained by the authors. All other used stand alone or command line software was free and publicly available: UCSF Chimera (https://www.cgl.ucsf.edu/chimera/download.html), KCOMBU (https://pdbj.org/kcombu/), anaconda was used as python environment (https://www.anaconda.com/products/distribution) with the free and open source available libraries (RDKit – https://www.rdkit.org/; OpenMM—https://openmm.org/ and sci-kit learn—https://scikit-learn.org/stable/).

The used proteins structure data were available from PDB (see Table 1 and Supporting Information Table SI3 for the for TR and TS_CRY_ PDB IDs). All TSMOD were computed starting from SMILES structures and are available in the Supporting Information Table SI2.

## Results and discussion

### Py-CoMFA model definition

**Fig. 2:**
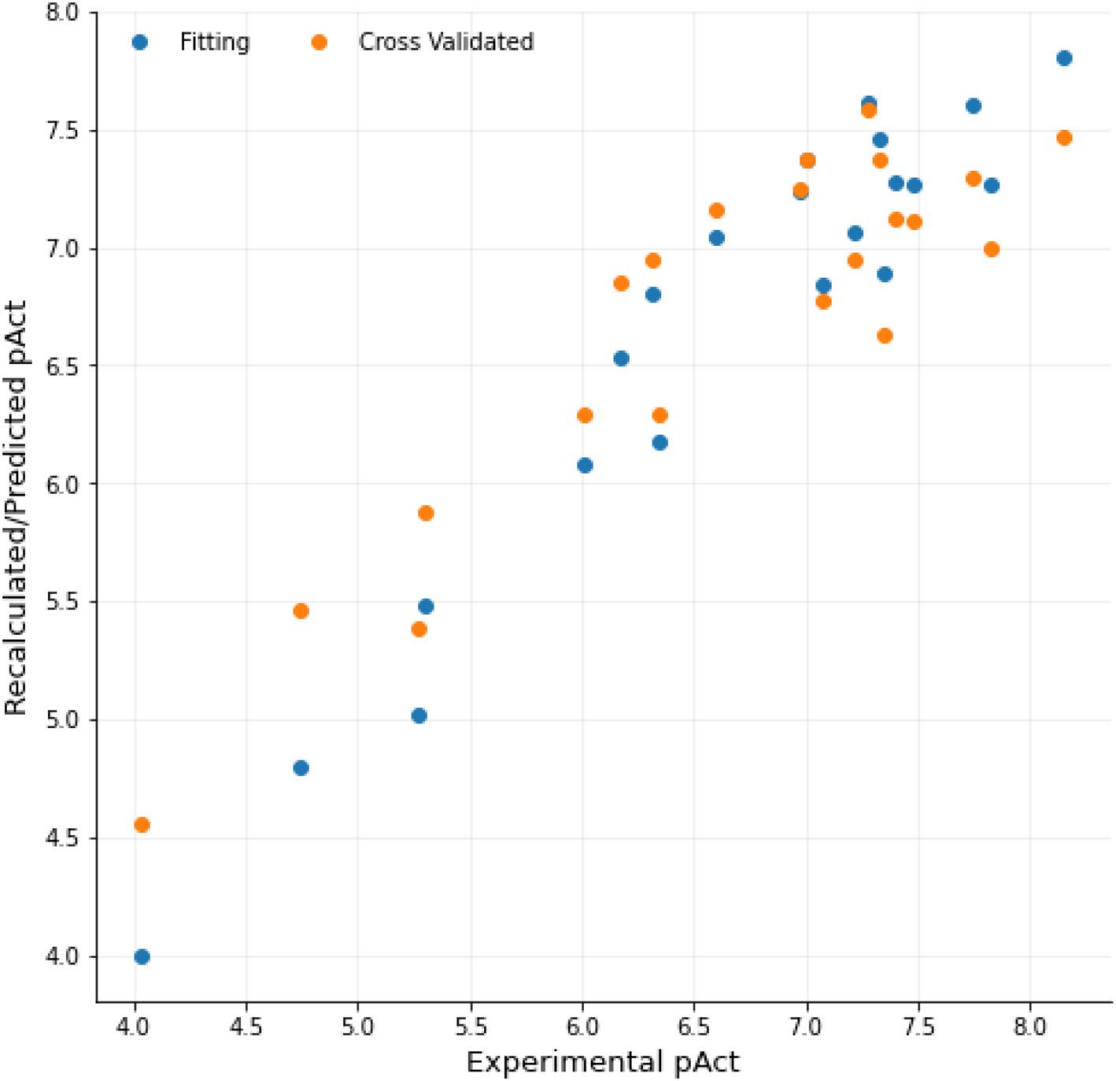
Model LB1 recalculated (blue dots) and internally predicted (orange dots, CV_LOO_) pIC_50_s versus experimental values (Table 2). pAct in the plot indicates the pIC_50_ as the plot was generated within 3d-qsar.com

Preliminary models built with the CV_LOO_ and the default settings showed satisfying statistical coefficients (*r*^2^ = 0.92, *q*^2^ = 0.63 for the BOTH-based Py-CoMFA model, Supporting Information Table SI11) with 2 principal components (PCs). Through the VPO protocol, more than 1300 3-D QSAR models were built to reach the optimized model characterized by *r*^*2*^ and *q*^*2*^ values up to 0.99 and 0.79, respectively. Among the VPO generated models, those obtained with a sp^2^ oxygen (O.2, model LB1, Fig. 2) and amidic nitrogen (N.am, model LB2) atom probes showed the highest statistical results (Table 2). Nevertheless, lower endowed statistical coefficients models LB3 and LB4, obtained with hydrogen and methyl probes, respectively, were also inspected as source of useful data for the subsequent graphical analysis. In general, the application of the VPO allowed to increase the *q*^2^ values in the range of 14–25%.

**Table 2:** VPO optimized Py-CoMFA models’ statistical results

Model	PC	Probe	*r*^2^	SDEC	*q*^2^_LOO_	SDEP _LOO_	*q*^2^_LSO_	SDEP _LSO_	*r*^2^_YS_	*q*^2^_YS_
LB1	2	O.2	0.92	0.30	0.79	0.50	0.76	0.52	0.70	− 0.05
LB2	3	N.am	0.96	0.22	0.74	0.55	0.71	0.58	0.87	− 0.01
LB3	2	H.P	0.92	0.30	0.72	0.57	0.67	0.60	0.81	0.18
LB4	3	C.3.H3	0.97	0.20	0.72	0.57	0.67	0.60	0.89	− 0.21

*PC* the optimal number of principal components, *Probe* atom probe used to calculate the MIFs, *r*^2^ conventional square correlation coefficient, *SDEC* standard deviation error of calculation, *q*^*2*^_*LOO*_ LOO cross-validation correlation coefficient, *q*^*2*^_*LSO*_ LSO cross-validation correlation coefficient—with 5 random groups and 100 iterations, *SDEP*_*LOO*_ LOO cross-validated standard error of prediction, *SDEP*_*LSO*_ LSO cross-validated standard error of prediction, *r*^*2*^_*YS*_ Y-scrambled conventional square correlation coefficient, *q*.^*2*^_*YS*_ Y-scrambled LOO cross-validation correlation coefficient

Models LB1 to LB4 were validated for both robustness and lack of chance correlation. In particular, cross-validation by either CV_LOO_ or CV_LSO_ methods showed a good level of model stability at different degrees of perturbation; Y-scrambling (YS) procedure verified the absence of models’ associated chance correlation, showing always lower *r*^*2*^_YS_ and *q*^*2*^_YS_ values than those obtained with unscrambled data (Table 2).

### Py-CoMFA model graphical interpretation

**Fig. 3:**
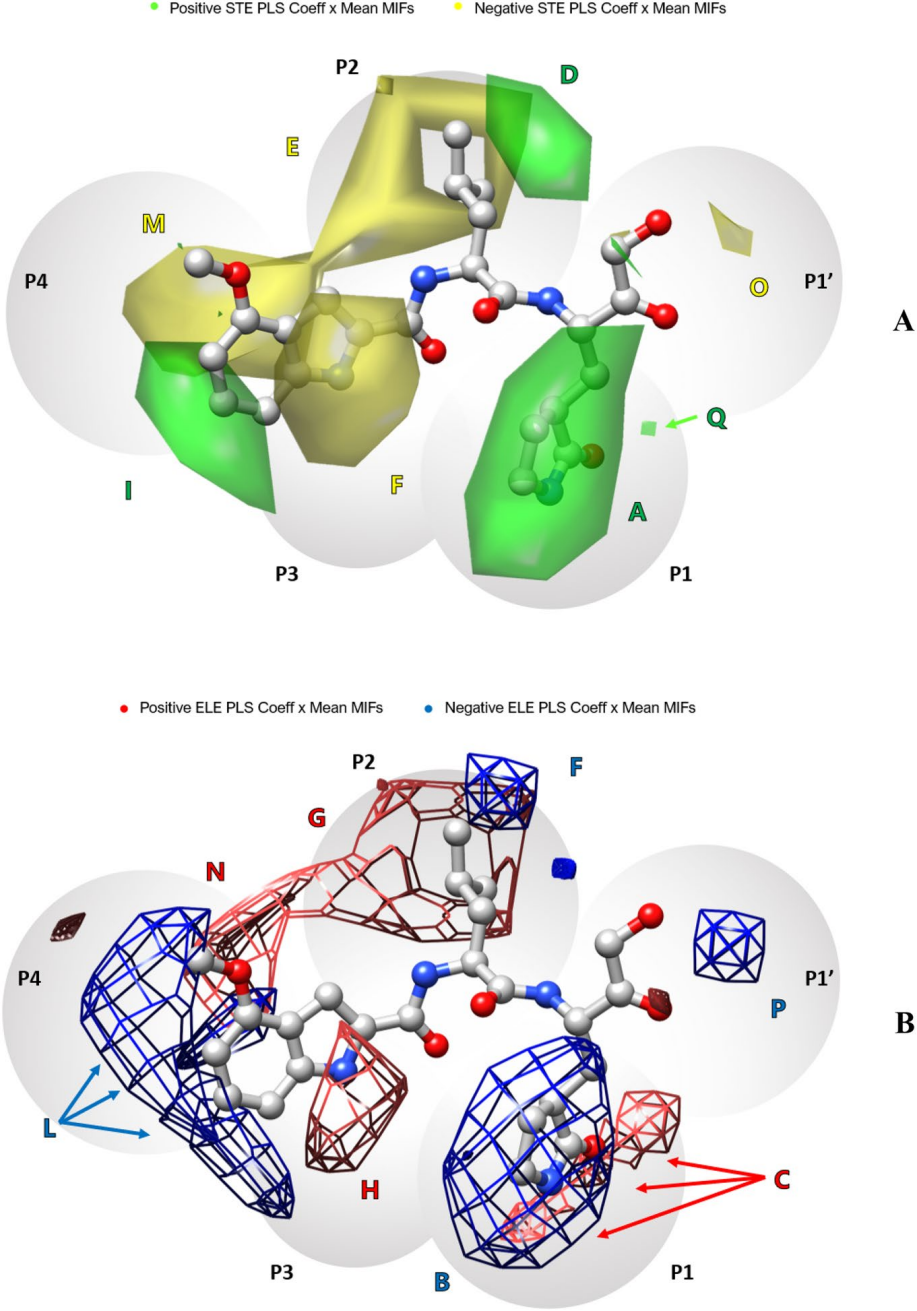
AAC steric (**A**) and electrostatic (**B**) plots of model LB1. The most potent compound **3** is shown (light gray). Green and yellow polyhedrons depict areas were increased or decreased steric bulk may favor biological activity, respectively. Red and blue polyhedrons indicate regions where electronically involved groups are predicted to positively or negatively contribute to the activity, respectively. Hydrogen atoms are omitted for the sake of clarity. These plots are generated by means of USCF Chimera

Evidence of the soundness of LB1 (Table 2) was proved by the analysis of the average activity contribution (AAC) contour plots, obtained via scalar product among average MIFs values and PLS coefficients (Coeffs). More detailed information was gained through the inspection of molecules’ activity contribution (AC) plots generated by the product of individual molecules’ MIFs values by Coeffs. AAC (Fig. 3, Supporting Information Figure SI1) and AC plots (Supporting Information Figure SI2) indicate either steric or electrostatic areas (in the shape of polyhedrons) around the molecules that are directly related to the associated biological response in a general or specific way, respectively. In order to better interpret the models, it is relevant to consider that TR compounds were SB aligned and consequently the 3-D QSAR grid box virtually embraced the substrate/inhibitor binding pocket. Therefore, the 3-D QSAR could be useful to correlate inhibitor molecular portions with the P1, P2, P3, P4 and P1’ corresponding substrate’s residues (Fig. 3) [78, 79].

Previous studies determined substrate specificity profile of SARS-CoV-2 M^pro^ and revealed it predominantly requiring a Gln in P1 position [80–82]. In this regards, a γ-lactam portion mimicking substrate’s Gln is recurrent in rationally designed compounds [27, 28, 31, 32]. Overlapped green and blue polyhedrons (panel A in Fig. 3 and panel B in Fig. 3, respectively) encompass P1 most potent compounds γ-lactam methylenes and amidic NH group (panel A and B of Supporting Information Figure SI1), suggesting a ligand should implement both a similar steric hindrance and the hydrogen bond donors (HD) properties. Similarly, red polyhedrons (C in Fig. 3 B) around the oxygen of the cyclic amide and the associated positive Coeffs indicated to further insert at least a hydrogen bond acceptor (HA) feature. At the P2 position, leucine is known to be the preferred residue [82], thus it is usually retained in designed inhibitors [25, 28, 31, 32]. However, other hydrophobic residues have been reported to influence the inhibitory potency [25, 29, 82]. In this region, the 3-D QSAR AAC plots indicate two different polyhedrons: a green polyhedron also slightly overlapping P1’ (D in Fig. 3 A) and a yellow one that expands towards P4 position (E in Fig. 3-A). AC plots (Supporting Information Figure SI2) were examined to evaluate the activity contribution of different ring systems in place of the leucine’s isobutyl moiety. In fact, in the modified peptide inhibitors, leucine replacement with a cyclohexylalanine (**10**, **13**, **15—**Supporting Information Figure SI2) or a phenylalanine (**7**, **9**, **12—**Supporting information Figure SI2) were observed to be related to larger green polyhedrons, indicating those residues to likely produce and extend the positive contribution of van der Waals favorable interactions with the M^pro^ S2 pocket (see below the SB results). The insertion of a benzothiazolyl ketone warhead in P1’ as in **17**, results in the orientation of the aromatic moiety towards P2 and likely boosts steric interactions within S2 (see below at the SB study) as correctly predicted by a larger green polyhedron (Supporting Information Figure SI2). Furthermore, AC plots (Supporting Information Figure SI2) reveal that the size of steric positive (green) or negative (yellow) polyhedrons (D and E in Fig. 3A) correlate with the P2 sidechain orientation (e.g. **4** and **5**—Supporting Information Figure SI2). The insertion of a rigid group as a saturated ring system or an unsaturated moiety could orientate the sidechain in such a way as to avoid E negative contribution but to exploit D positive one.

Regarding electrostatic contributions, around P2 position two polyhedrons with opposite contributions overlapped the steric D and E ones. Respectively, a blue polyhedron (F in Fig. 3B) suggested to favor HA substituents while, on the contrary, a red polyhedron that extended towards P4 (G in Fig. 3B) indicated to increase HD groups in that area to favor biological activity.

In P3, AAC plots and AC plots show a yellow polyhedron (F in Fig. 3A) associated with the side chains of valine, leucine and O-tert-butyl-threonine (**1, 6, 8–13, 18–21**—Supporting Information Figure SI2) as well as a red electrostatic polyhedron (H in Fig. 3B) around the bulkier groups, labeling steric and HD features as undesirable for the potency. P3-P4 capping moieties can exhibit a wide variety of functional groups which could magnify binding affinity and modulate selectivity and potency of the inhibitors. Unfortunately, these regions were not covered by the TR molecules, which predominantly displayed an indole group (**3**, **15**, **16**, **17**) and benzyloxycarbonyl (CBZ) group (**2**, **5–13**). These groups were related to a green polyhedron (I in Fig. 3B) and many blue polyhedrons (L in Fig. 3A) that emphasized the importance to focus on this portion to capture additional hydrophobic and hydrogen bond contributions and to improve drug-like properties. Regarding P4 position, yellow polyhedrons (M in Fig. 3A) covered the cyclohexylglycine residue of lesser potent compounds **19** and **20**: larger yellow polyhedrons in the corresponding AC plots (Supporting Information Figure SI2) indicated that steric hindrance in that area should be avoided and clarified the low potency associated to these compounds. Red polyhedrons overlapping M (N in Fig. 3B) suggest avoiding HD features and eventually prefer HA ones to enhance bioactivity, while maintaining a reduced steric hindrance. In P1’, the explored chemical warheads are α-ketoamides, ketones and aldehydes. AAC plots and more specifically AC plots associate negative contributions to α-ketoamide warheads (yellow polyhedron O in Fig. 3A) and characterized them as penalizing for the activity (**5, 6 14, 18, 19, 20**—Supporting Information Figure SI2). Nonetheless, the lack of training set chemical warheads diversity could elucidate the poor performance of the α-ketoamides warheads instead of aldehydes, that were designed at the early stages of lead optimization, to retain the aldehyde in P1’ and to slightly replace other positions (P1—P4) [25, 29]. For these reasons, preferring an aldehyde instead of any other warhead would be a workaround choice, due to practical-synthetic requirements. An ideal warhead should be designed to better mimic the scissile moiety of the peptidyl substrate or the tetrahedral intermediate, it should ensure a proper orientation within the subsite and be stabilized by the interactions with oxyanion hole residues of the protease (Cys145 and Gly143). A noteworthy suitable warhead, the benzothiazolyl ketone unit of compound **17**, showed a different orientation compared to the others and appears to establish favorable steric interactions towards P2 (Supporting Information Figure SI2). Regarding electrostatic contributions, a blue polyhedron that encounters O (P in Fig. 3-B) associated with negative Coeffs suggests to avoid HD groups to enhance potency.

#### Py-ComBinE model definition

**Fig. 4:**
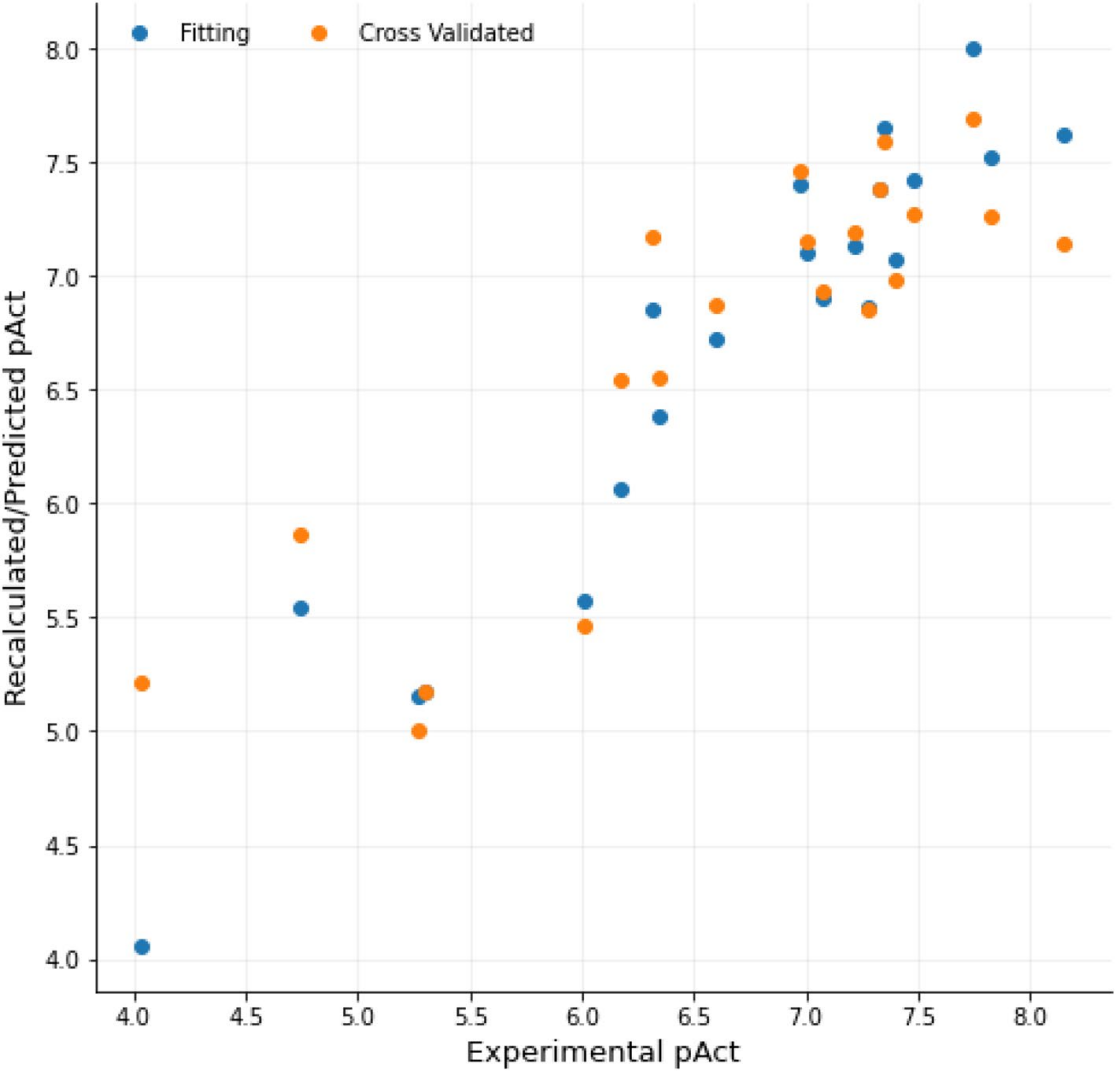
Recalculated (blue dots) and CV_LOO_ predicted (orange dots) pIC_50_ values versus the experimental activities by model SB1_SAFS_ (Table 3). pAct in the plot indicates the pIC_50_ as directly generated by 3d-qsar.com. The plot was generated within 3d-qsar.com

15 preliminary models were built (SB1-SB15, Table SI12) and among them model SB7 (STE.HB) was endowed with the highest statistical results (*r*^2^ = 0.91, *q*^2^ = 0.69). Nonetheless, in order to investigate the whole variety of key/lock interactions type (STE, ELE, DRY and HB), it was decided to focus on the STE.HB.ELE.DRY model that by means of the SAFS algorithm was optimized into a Py-ComBinE model endowed of *r*^2^ and *q*^2^ statistical values of 0.90 and 0.77, respectively (model SB1_SAFS_, Table 3, Fig. 4). For completeness, the SAFS algorithm was also applied to other combinations interactions leading to worse or comparable results (SB2_SAFS_-SB3_SAFS_, Table 3). Thus, to disclose as much as possible data and reduce redundancy, model SB1_SAFS_ was herein inspected and discussed.

**Table 3:** SAFS optimized Py-ComBinE models’ statistical results

Model	Interactions	LOO				LSO				YS	
		*r*^2^(PC)	SDEC	*q*^2^	SDEP	*r*^2^(PC)	SDEC	*q*^2^	SDEP	*r*^2^	*q*^2^
SB1_SAFS_	STE.ELE.DRY.HB	0.90 (2)	0.33	0.77	0.51	0.91 (2)	0.33	0.74	0.54	0.07	0.54
SB2_SAFS_	STE.DRY.HB	0.93 (3)	0.28	0.79	0.48	0.91 (3)	0.31	0.78	0.50	0.16	0.25
SB3_SAFS_	STE.HB	0.92 (3)	0.30	0.78	0.50	0.91 (3)	0.31	0.75	0.52	0.39	-0.68

PC: optimal number of principal components; Fields: field or combination of fields used to calculate the interactions; *r*^2^: conventional square correlation coefficient; SDEC: standard deviation error of calculation; *q*^2^_LOO_: LOO cross-validation correlation coefficient; *q*^2^_LSO_: LSO cross-validation correlation coefficient—with 5 random groups and 100 iterations; SDEP_LOO_: LOO cross-validated standard error of prediction; SDEP_LSO_: LSO cross-validated standard error of prediction; *r*^2^_YS_: Y-scrambled conventional square correlation coefficient; *q*^2^_YS_: Y-scrambled LOO cross-validation correlation coefficient

To assess model’s internal predictive power and robustness, LOO and LSO methods were chosen for cross-validation, obtaining *q*^2^ values of 0.77 and 0.74 respectively, with only 2 principal components. These results suggested a good internal predictability of the model. Y-scrambling (YS) results guaranteed that the correlation between the biological data and the independent variable did not result from a chance correlation.

#### Py-ComBinE model graphical interpretation

**Fig. 5:**
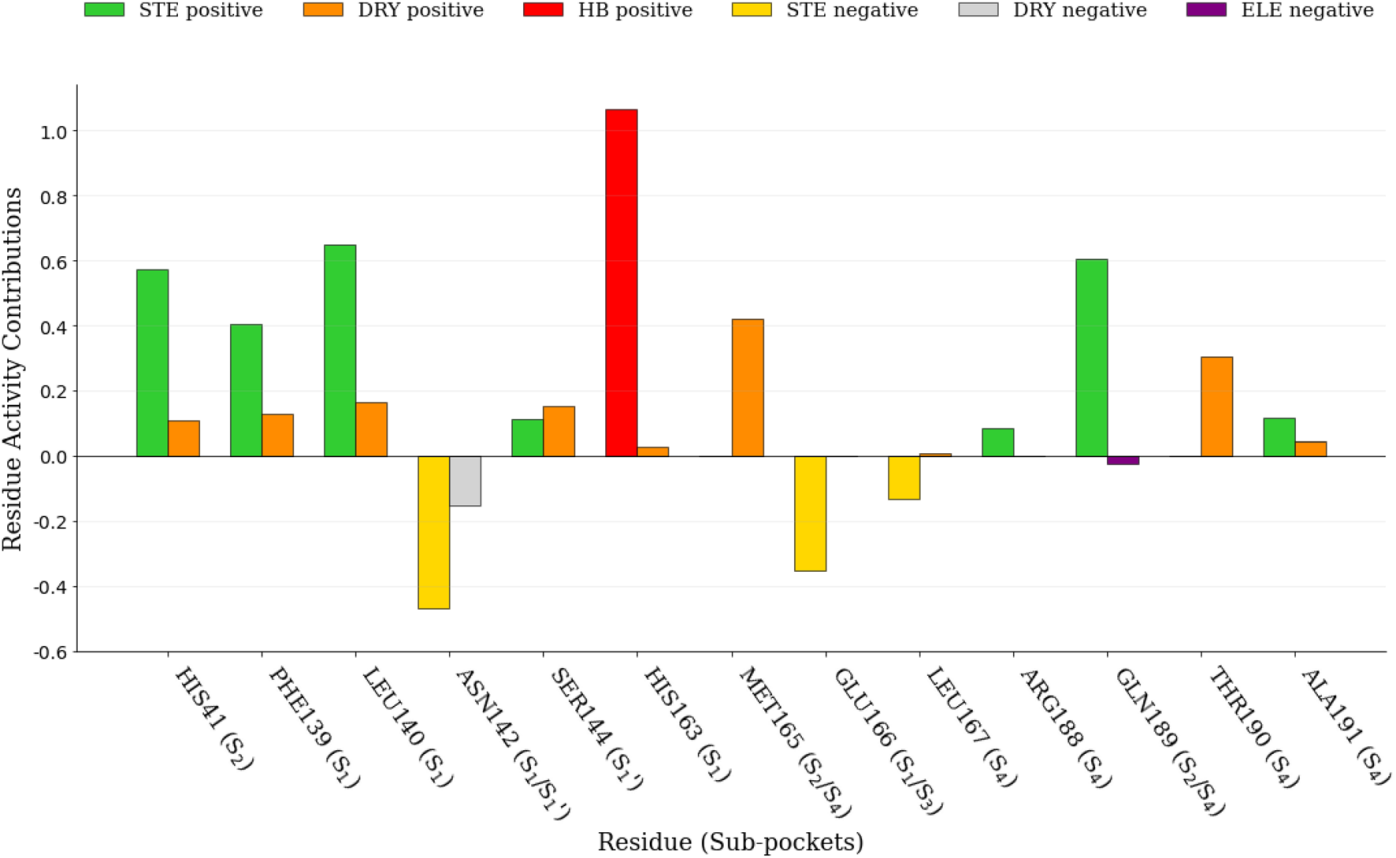
MRAAC plot of model SB1_SAFS._ The most relevant ligand/per-residue positive or negative energetic interactions are reported: steric (STE), electrostatic (ELE), desolvation (DRY) and hydrogen bond (HB). Aside the residue numbers in bracket are reported the indication of the enzyme’ pocket to which each residue belongs

**Fig. 6:**
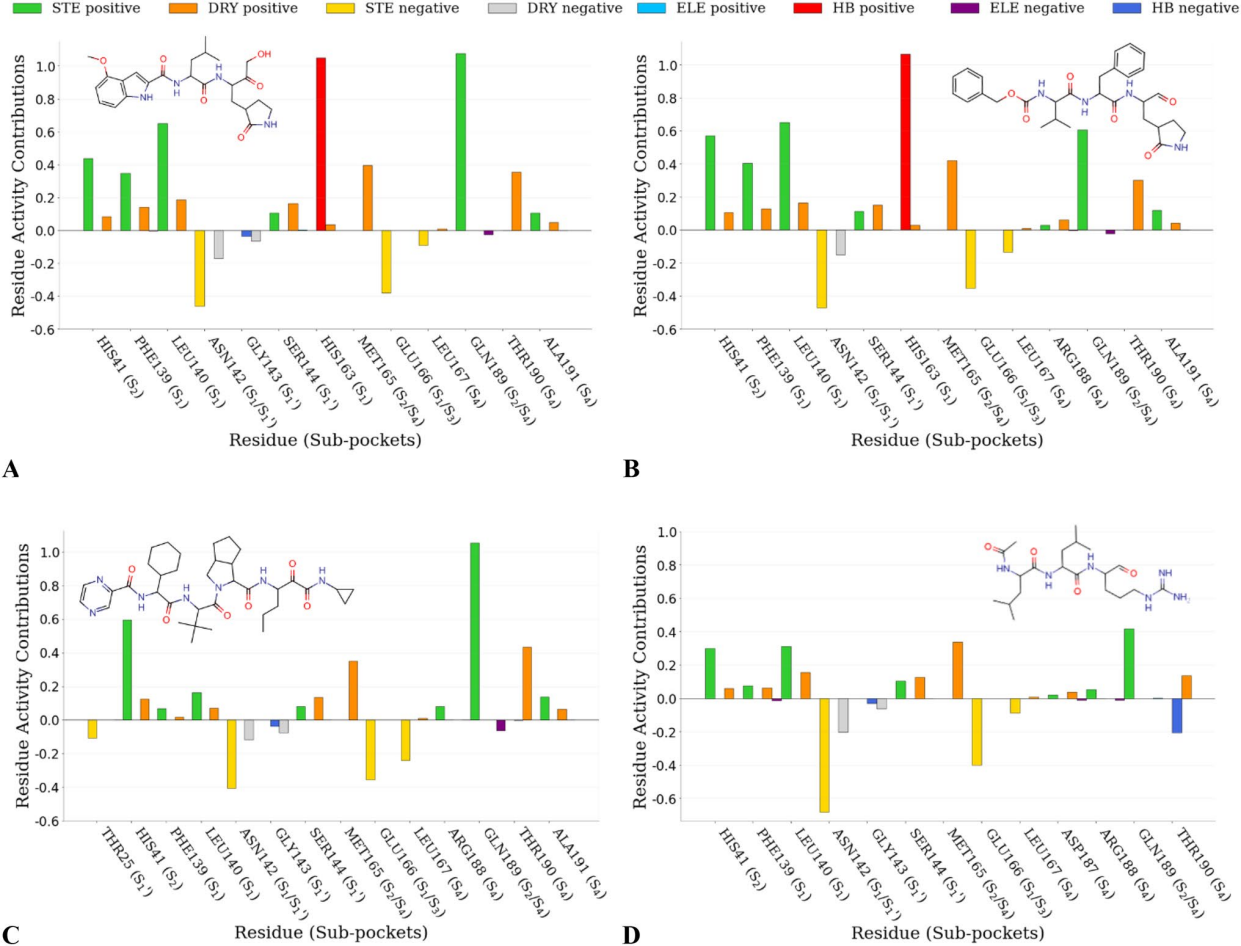
MRAC plots of the two most active TR compounds 3 (**A**) and 9 (**B**) and the two least active TR compounds 20 (**C**) and 21 (**D**) derived by model SB1_SAFS_. The most relevant ligand/per-residue positive or negative energetic interactions are reported: steric (STE), electrostatic (ELE), desolvation (DRY) and hydrogen bond (HB). Aside the residue numbers in bracket are reported the indication of the enzyme’ pocket to which each residue belongs

Similarly to the above-reported 3-D QSAR analysis, the COMBINE models were visually inspected by means of two types of plots: the molecule-residue average activity contribution (MRAAC), obtained by multiplying the average molecule-residue interaction values by PLS coefficients (Fig. 5) and the molecule-residue activity contribution (MRAC) plot, representing the scalar product between the individual molecule-residue interaction values multiplied and PLS coefficients (Fig. 6). These plots correlate training set molecules with biological activity and can aid to individuate the protein fragments which are more involved in modulating the overall ligand/protein interaction. The global importance of the interactions can be understood similarly to the aforementioned 3-D QSAR models: the positive values are directly correlated with a favorable interaction and consequently enhanced bioactivity; conversely, the negative values correlate with decreased biological affinities.

Inspection of the steric MRAAC plots indicated His41, Phe140, Leu141 and Gln189 as those residues playing a major role in modulating the overall inhibitory potency, therefore the interaction with these residues should be retained, while low negative values were associated with Asn142, Glu166 and Leu167 residues, specifying that the ligands’ interaction with them should be lowered to increase the potency (Fig. 5).

Regarding the residues mainly involved in hydrogen bonds, only His163 turned out to be responsible for a positive ligand–protein interaction, in fact, a hydrogen bond is established for the two most active molecules **3** and **9**, while the least potent molecules lack of any hydrogen bond (Fig. 6). A favorable desolvation interaction was found associated to His41, Phe140, Leu141, Ser144, Met165 and Thr190 residues, whereas a negative value was only related to Asn142 (Fig. 5). Electrostatic interactions were the less represented by the model, which identified only a tiny negative contribution associated with Gln189.

### Combination of 3‑D QSAR and COMBINE

**Fig. 7:**
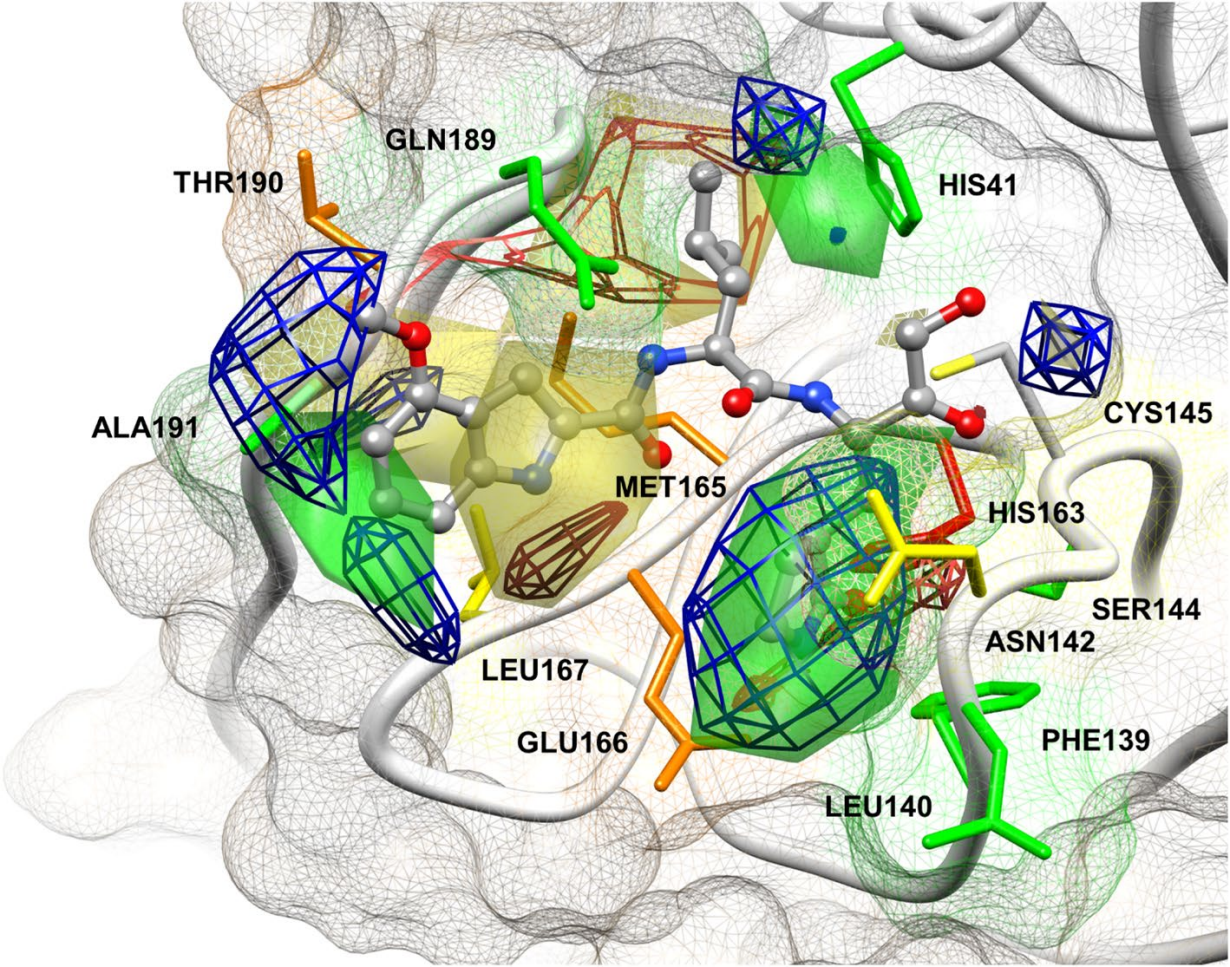
Graphical depiction of AAC and MRAAC plots in the binding site of compound **3** (gray)—M.^pro^ minimized complex (PDB code = 6XHM). Residues are colored depending on their higher activity contribution: green—STE positive, yellow—STE negative, red—HB positive, orange—DRY positive (see legend in Fig. 5). The image was prepared through USCF Chimera

Residues associated with the most relevant activity contributions from COMBINE analysis (Fig. 5) were highlighted in M^pro^ binding site and overlapped with the 3-D QSAR maps (Fig. 3) to have a straightforward graphical view of the results of each technique (Fig. 7). Despite the different approaches of the employed methods (LB and SB), their results and indications were in good agreement and synergistically strong supported each other. P1 moiety is deeply embedded and stabilized in S1 sub-pocket and according to the above discussed results (Fig. 5, panel A and B of Fig. 6), the strongest hydrogen-bonding interaction was due to the bond between the Gln mimetic group carbonyl oxygen in P1 and His163 as part of S1 sub-pocket. The positive green polyhedron (A in Fig. 3 A) supported the favorable steric contributions of Leu141 and Phe140 (Fig. 5), located at the bottom of the cleft (Fig. 7). As a matter of fact, the low potent compounds (i.e. **20**, **21**) lacked of these crucial interactions (panels C and D of Fig. 6). The buried S2 sub-pocket usually accommodated P2 substrate leucine side chain, but showed to be large enough to tolerate bulkier alkyl or aryl moieties to maximize van der Waals interactions [13, 82]. A green polyhedron in this area (D in Fig. 3-A) corroborates the high positive contribution associated with His41 (Fig. 5). The less structured S3 and S4 sub-pockets defined by flexible loops can rearrange upon ligand binding and accommodate groups of various size and nature [13, 15, 82]. The yellow polyhedron around P3 (F in Fig. 3 A) matched with the negative contribution associated with Glu166 in the shallow and solvent-exposed S3 pocket (Fig. 5). In S4 small hydrophobic pocket, steric green and yellow polyhedrons (I and M in Fig. 3 A) fitted well with the results of SB analysis as the interaction of ligands with Gln189 and Ala191 positively contribute to the activity while the interaction with Leu167, at the bottom of the pocket, should be avoided (Fig. 5).

In S1’, Asn142 negative activity contributions were confirmed by a yellow polyhedron around P1’ bulky warheads (O in panel A of Fig. 3), while a small green polyhedron (Q in panel A of Fig. 3) confirmed the favorable contribution of interactions with Ser144 (Fig. 5). The red polyhedron in the oxyanion hole (C in panel B of Fig. 3) was likely due to the hydrogen bonding interactions established anticipating the Cys145 nucleophilic attack and the consequent covalent adduct formation.

A deep analysis of MRAC plots (Fig. 6) led to the following observations. His41 positive activity contribution was higher when interacting with electron-rich and bulky P2 side chains (panel B of Fig. 6) as compared to smaller moieties (panel D of Fig. 6): these data were in good agreement with the corresponding AC plots (Supporting Information Figure SI2). Gln189 positive contribution was increased by cyclic leucine mimetic moieties in P2 (panel C of Fig. 6). On the contrary, Leu167 negative contribution was bigger when interacting with some of the less potent compounds (panel C of Fig. 6) that fitted into S4 and were surrounded by big negative polyhedrons in the corresponding area in AC plots. About P1’, compounds with a bulky and flexible moiety also interacted with Thr25, which negatively contributed to the potency (panel C of Fig. 6).

### 3-D structure–activity relationship

**Fig. 8.**
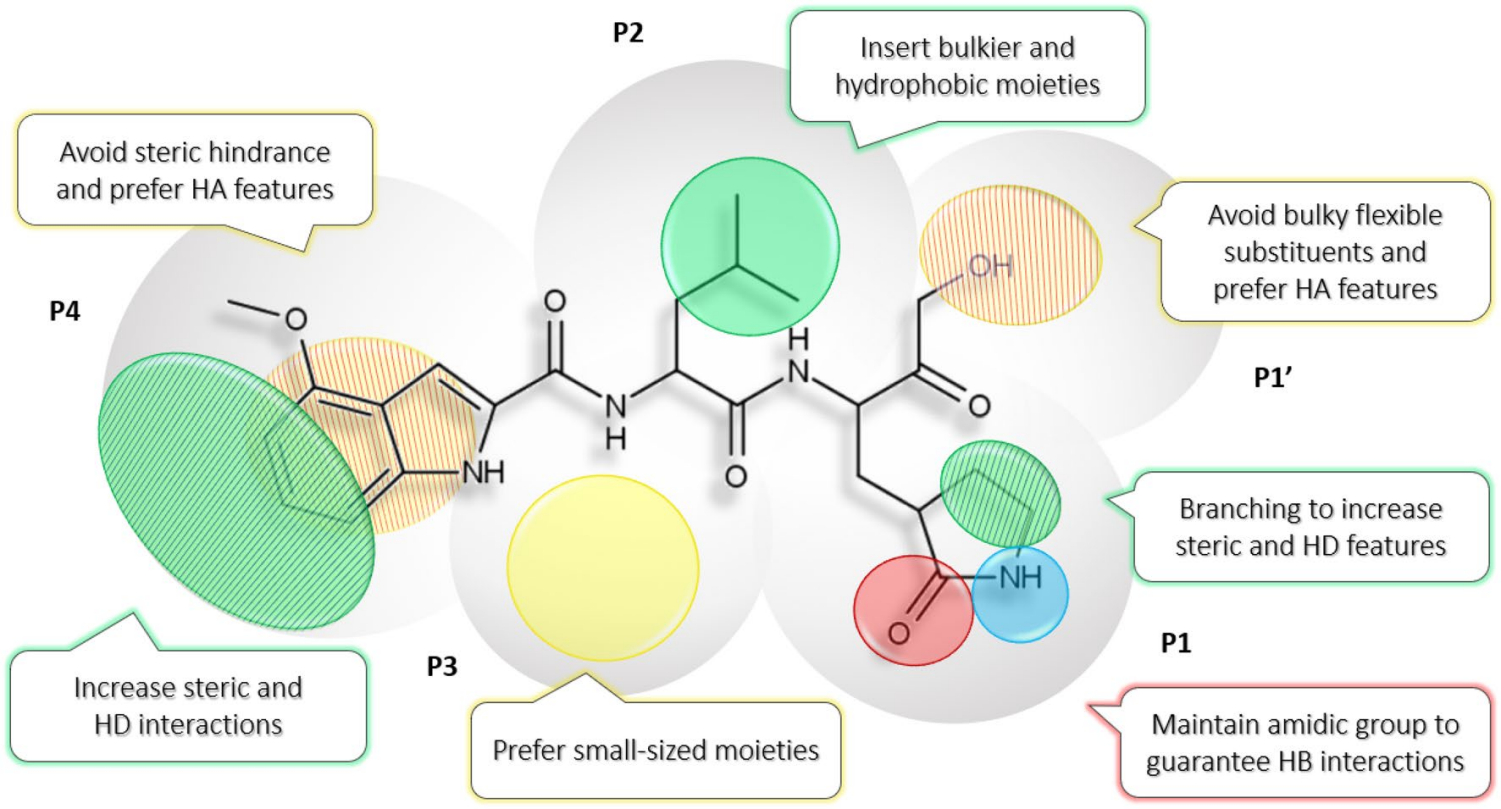
3-D SAR derived model for M.^pro^ inhibitors. The most potent TR compound **3** is used as template. Circles are color-coded to represent the main associated steric (favorable green, unfavorable yellow) and HB (HD blue, HA red) features. Striped two-colored circles account for two features together

Given the good agreement among the 3-D QSAR and COMBINE models, comprehensive 3-D structure–activity relationship (SAR) rules could be derived (Fig. 8) by combining the above graphical analysis. This led to derive a unique SAR as a tool to drive the design of potential new anti-coronavirus agents. Moreover, considering the covalent to reversible structures conversion the herein models could be used to design both types of inhibitors regardless of the warhead reactivity.

In P1’ position, neither flexible nor bulky substituents seemed to be the best choice to reduce negative contributions and interaction with Asn142. This could be avoided by using less bulky moieties than α-ketoamides, less reactive than aldehydes or that eventually orientate towards S2 sub-pocket, as for instance the benzothiazolyl ketone unit of compound **17**. Moreover, as highlighted above, this moiety should not bear an HD feature. In P1 position, the Gln mimetic γ-lactam moiety remains the preferred to ensure the steric interactions with Leu141 and Phe140 but, to further increase van der Waals interactions, the ethylene bridge could be properly branched while HA interactions (the hydrogen bond between the amide portion and His163) should be retained. Concerning P2 position, a bulky hydrophobic moiety like phenylalanine could be better tolerated than the substrate-like leucine since it establishes π-π stacking interactions with His41, although constraining leucine in a cyclic unit could improve the interaction with Gln189. About P3-P4 capping groups, the indole group ensures the steric and electrostatic interactions highlighted by either AAC and MRAAC plots: positive interactions with the shallow residues Gln189 and Ala191 while avoiding the penalizing ones with residues Glu166 and Leu167. Alternatively, substitutions on indole or CBZ groups could intensify hydrophobic interactions and add up hydrogen bonding interactions. In P3, small-sized groups are preferred over bulky ones and in P4 extensive steric and HD features should be avoided.

### 3‑D QSAR and COMBINE predictive ability and their combination

#### Py-CoMFA model predictive ability evaluation

Considering the satisfactory models’ internal validation, the above described TS_MOD_ and TS_CRY_ were used to evaluate model LB1 predictive ability, which was promptly confirmed by low errors of prediction in the range 0.01–2.84, low absolute average error of prediction (AAEP) of 0.93 and a standard deviation error of prediction (SDEP_PRED_) of 1.12 (Table 4, Supporting Information Figure SI3 and SI4). Concerning TS_MOD_, LB1 overpredicted low potent compounds and underpredicted only a few high potent compounds (Supporting Information Figure SI3). Likely, overprediction was due to the intrinsic alignment assumption that less potent compounds adopt conformations that are comparable to those of potent compounds. On the contrary, TS_CRY_ predictions trend was better reproduced by LB1 (Supporting Information Figure SI4).

**Table 4:** LB1 and SB1SAFS models’ predictive ability. SDEPPRED, AAEP values are reported. Models’ consensus predictivity abilities are also included (see main text)

TEST SETS	LB1			SB1SAFS			CONSENSUS		
	Total	TSMOD	TSCRY	Total	TSMOD	TSCRY	Total	TSMOD	TSCRY
SDEPPRED	1.12	1.16	1.06	1.11	0.87	1.40	0.93	0.85	1.05
AAEP	0.93	1.03	0.78	0.88	0.68	1.16	0.75	0.72	0.82
Min	0.01	0.07	0.01	0.02	0.05	0.02	0.01	0.01	0.01
Max	2.84	1.97	2.84	2.60	2.60	2.52	2.56	2.05	2.56

*SDEPPRED* standard deviation error of prediction, *AAEP* average absolute error of prediction, *Min and Max* indicate the range of absolute error of predictions

#### Py-ComBinE model predictive ability evaluation

The external predictivity of SB1_SAFS_ was verified through the TS_MOD_ and TS_CRY_ molecules and gave satisfying absolute errors of prediction in range 0.02–2.60, AAEP of 0.88 and SDEP_PRED_ of 1.11 (Table 4, Supporting Information Figure SI5 and SI6). SB1 model was more able than LB1 to reproduce TS_MOD_ correct activity trend and returned lower AAEP and SDEP_PRED_ values (Table 4, Supporting Information Figure SI5). On the contrary, SB1 was more unreliable on TS_CRY_ predictions, especially with non-covalent subgroup (Supporting Information Figure SI6). Nevertheless, the fact that TS_CRY_, compiled with experimental poses, and TS_MOD_, compiled with modeled poses, had comparable errors of predictions (Table 4) further supports the reliability of the models predictive ability.

#### 3‑D QSAR and COMBINE predictive ability consensus

**Fig. 9:**
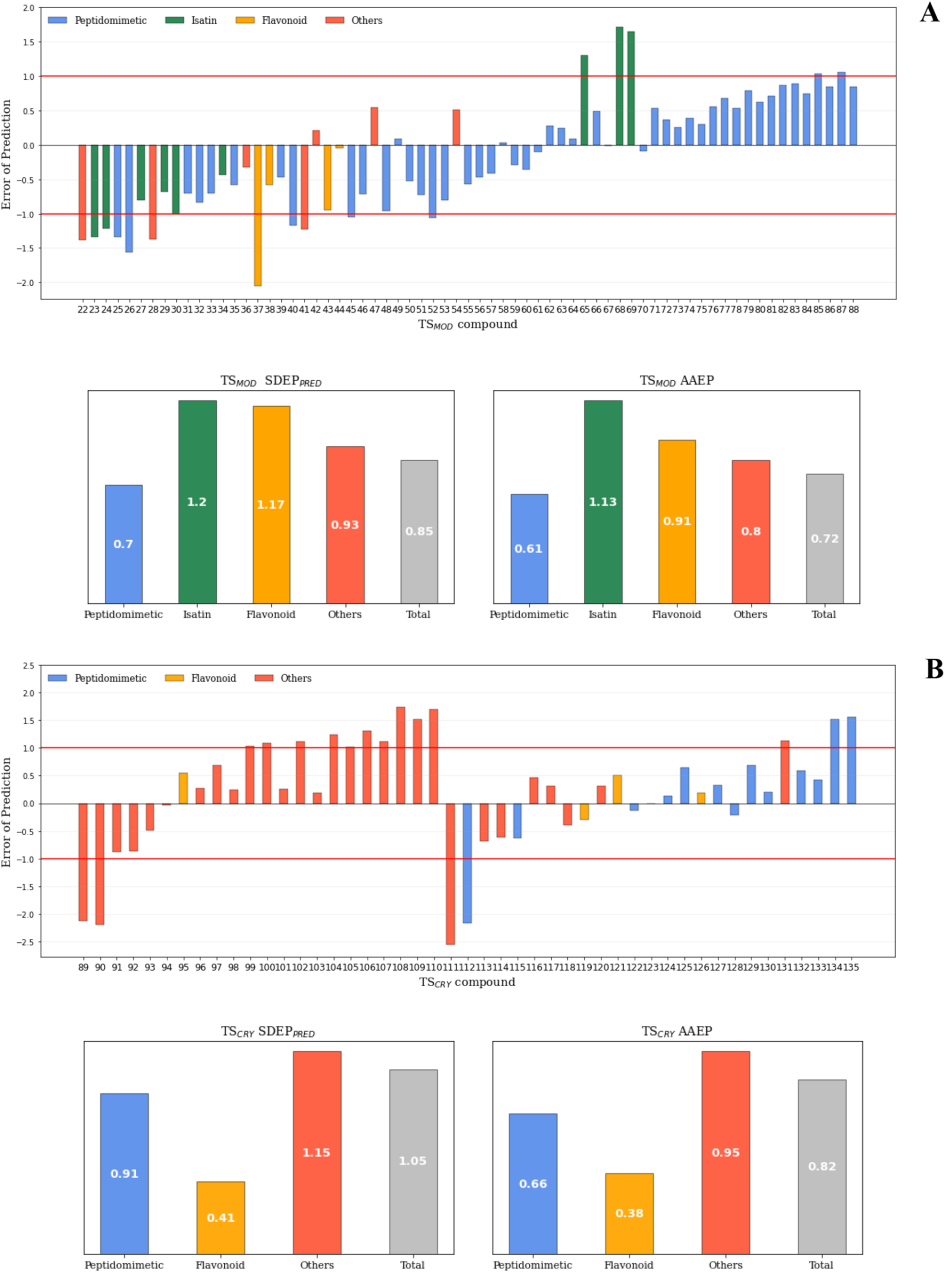
TSMOD (**A**) and TSCRY (**B**) consensus model’s errors of prediction, SDEPPRED and AAEP, divided by scaffolds

The good trend of fitting and internal validation results was confirmed by globally low absolute average error of predictions (AAEP) and standard deviation error of prediction (SDEP_PRED_) of either LB and SB techniques for both TS_OD_ and TS_CRY_ external sets (Table 4). To merge the LB1 and SB1 models’ predictive power, a linear regression model was derived using the predicted activities of TS_MOD_ and TS_CRY_ of both models to weight each model importance. Interestingly and somehow expected, the consensus model returned lower SDEP_PRED_ and AAEP values of 0.93 and 0.75, respectively (Table 4, Fig. 9).

Setting to 1.0 pIC_50_ as the generically acceptable arbitrary threshold value of absolute error of prediction (AEP, Fig. 9), the percentage of low error predicted compounds by consensus model was 75% (79% TS_MOD_ and 68% TS_CRY_).

Among the underpredicted compounds, **68** (pIC_50_ = 7.33) and **108** (pIC_50_ = 7.60) showed the highest errors of prediction (~ 1.7 pIC_50_). AC and MRAC plots were analyzed and revealed that compound **68** did not fill S1 pocket and consequently the diminished van der Waals and HB interactions with Phe140, Leu141 and His163 residues led to underestimate its pIC_50_. On the other hand, **108** had negative steric interactions with Asn142 in S1’, Glu166 in S3 and lacked favorable HB interaction with His163 in S1 and therefore a lower predicted pIC_50_ returned (data not shown).

Among the overpredicted compounds, the two low potent inhibitors **90** (pIC_50_ = 4.27) and **111** (pIC_50_ = 3.86) showed the highest errors of prediction (> 2.0 pIC_50_). Compared to the TR molecules, **90** and **111** have different *pose* and chemical scaffolds and lack P1’ and P4 but largely occupy S1 and S2 pockets, thus leading to overcalculated van der Waals and HB interactions with Phe140, Leu141, His163 and His41 (data not shown) accounting for their overpredicted pIC_50_ values.

**Fig. 10:**
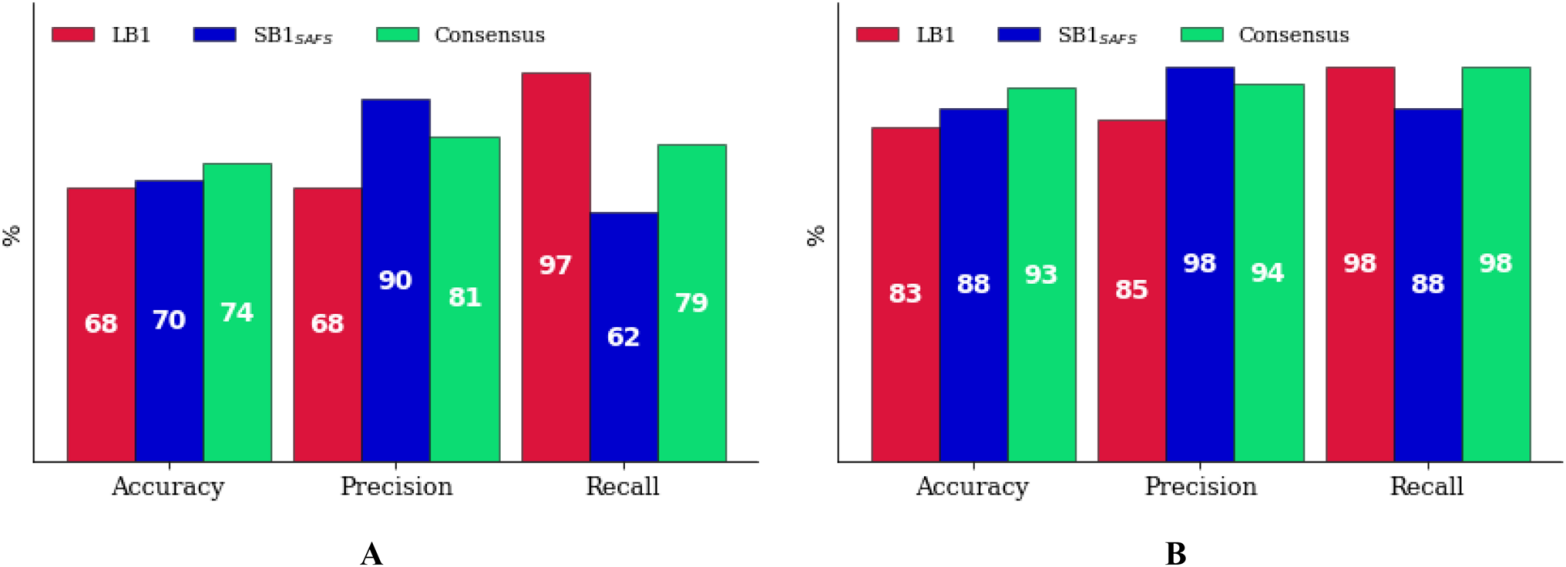
Classification metrics for LB1, SB1_SAFS_ and Consensus models before (**A**) and after (**B**) assessing the AD

In addition, applying a threshold value of 1 μM IC_50_ (6.0 pIC_50_), the models classification performance, in terms of accuracy, precision and recall metrics [83] (Supporting Information Figure SI7), was also inspected (panel A of Fig. 10). In both test sets predictions, the consensus model showed balanced performances and was able to overcome some inaccurate results of the single LB1 and SB1_SAFS_ models.

Noteworthy, the consensus model successfully classified as true positive or true negative returning the highest accuracy value of 74% for all TS_MOD_ and TS_CRY_ molecules, overcoming the LB1 low accuracy (68%). Moreover, the consensus model showed high precision (81%) and recall (79%), that respectively designate the correctly classified-experimentally active compounds among all the active-labelled compounds (positive predicted value) and the correctly classified-experimentally active compounds among all the experimentally active ones (true positive rate). Regarding recall, the analysis of the false-negative molecules revealed most of them to have a considerably different scaffold from any of the TR compounds as evinced by low Tanimoto similarity index values in the 0.15–0.35 range (Supporting Information Table SI8 and Table SI9).

In order to provide more reliable predictions and define a model chemical space coverage, its applicability domain (AD) was defined by means of a *k*-nearest neighbors (*k*-NN) approach (see Applicability domain definition in Supporting Information) [84–86]. Application of the AD reduced the test set from 114 to 60 compounds leading to a definite improvement of both models’ predictive statistical parameters (SDEP_PRED_ = 0.68, AAEP = 0.57) and classification performances (panel B of Fig. 10) being 90% of them were predicted with an AEP < 1.0 pIC_50_.

## Conclusion and perspectives

The ongoing COVID-19 pandemic sorely stretched global public health to the limit. Despite the rapidly-developed vaccines have been crucial for weakening the most severe implications of the disease and for reducing the probability of infection, emerging variants and the resulting increment of breakthrough infections demand the urgent need for specific medications against SARS-CoV-2. Since the first SARS-CoV outbreak in the early 2000s, the M^pro^ has been gaining more and more attention for its key role in viral replication and transcription, thence repurposed drugs and new rationally designed compounds fulfilled M^pro^ inhibition strategy.

In this study, consistently selected M^pro^ inhibitors were used to develop robust and predictive 3-D QSAR and COMBINE models that could greatly assist in rapid virtual screenings and in the discovery of new leads. Besides, the convergence of 3-D QSAR contour plots and COMBINE histograms analysis gave useful insights in characterizing relevant features to design new inhibitors by maximizing ligand/protein interactions.

Graphical inspection of results led to depict a three-dimensional (3-D) structure analysis relationship (SAR) scheme that could be used as a guideline for the design and discovery of new potential M^pro^ inhibitors, saving both time and financial resources to fight SARS-CoV-2.

As discussed, the predictive ability of the models gave convergent statistical values and confirmed models’ feasibility on either co-crystallized (TS_CRY_) and non-crystallized (TS_MOD_) compounds. Moreover, once assessed the AD of the models, higher predictive performances metrics were obtained.

Upon this project completion, a new report disclosed to the discovery, characterization and FDA emergency use authorization (EUA) of nirmatrelvir, an orally bioavailable M^pro^ peptidomimetic covalent inhibitor, and some relevant analogs. Remarkably, the 3-D SAR above-described were in good agreement with the strategy followed by Owen et al. [40] In fact, they opted for a benzothiazolyl ketone or nitrile unit in P1’ to remove HD; they maintained the native glutamine mimicking unit in P1; they broadened steric hindrance in P2 constraining the leucine native residue, concurrently increasing steric interactions and removing the HD; finally, they chose small-medium sized units in P3 and increased HA in P4.

In conclusion, the LB and SB procedure herein described represent a useful tool to design potential new chemical entities as M^pro^ inhibitors to study as broad anti-coronavirus agents.

## Supplementary Information

Below is the link to the electronic supplementary material.

Supplementary file1 (PDF 1988 kb)

Supplementary file2 (XLSX 491 kb)
