# Supplementary material for: Ligand-based and structure-based studies to develop predictive models for SARS-CoV-2 main protease inhibitors through the 3d-qsar.com portal
Source: J Comput Aided Mol Des. 2022 Jun 18;36(7):483–505. doi: 10.1007/s10822-022-00460-7 (PMC9206107; doi:10.1007/s10822-022-00460-7)
Supplement: Supplementary file 1 — Supplementary file1 (PDF 1988 kb) [file 10822_2022_460_MOESM1_ESM.pdf]

Supplementary Information

Ligand-Based and Structure-Based Studies to Develop Predictive Models for SARS-CoV-2 Main Protease Inhibitors Through the 3d-qsar.com Portal

Eleonora Proia,[ς] Alessio Ragno,[¥] Lorenzo Antonini,[ς] Manuela Sabatino,[ς] Milan Mladenovič,[§] Roberto Capobianco[¥,≠] and Rino Ragno[ς,*]

[ς]Rome Center for Molecular Design, Department of Drug Chemistry and Technology, Sapienza University of Rome, P.le Aldo Moro 5, 00185 Rome, Italy

[¥]Dept. of Computer, Control and Management Engineering "Antonio Ruberti", Sapienza University of Rome

[§]Kragujevac Center for Computational Biochemistry, Department of Chemistry, Faculty of Science, University of Kragujevac, Radoja Domanovića 12, 34000 Kragujevac, P.O. Box 60, Serbia

[≠]Sony AI

Dataset compilation.

At the beginning of the project, 77 M^{pro} inhibitors were collected from literature [1-15]: the 21 co-crystallized ones were used to compile the training set (TR, Table 1, Supporting Information Table SI1), while the 56 non-complexed inhibitors were left out and used as first external modeled test set (TS_{MOD}). For compounds with multiple reported IC_{50} values (**2**, **18**, **19**, **45**, **95**, **119**) the average value was used. Due to the likely pro-drug behavior, the bisulfite compound **2** was modeled to pre-covalent active aldehyde, as the putative reversible like inhibitory species [16]. For the same reason, bisulfite adducts listed in ref. [10] were not included. Compound **17** IC_{50} value was calculated from K_i according to Cheng-Prusoff [17]. During the investigation, the 15 compounds listed in ref. [18] were removed from the TS_{MOD} due to conflicting biological results reported in recent reports [19, 20]. On the other hand, other 32 inhibitors taken from more recent literature [21] were added, reaching a number of 73 TS_{MOD} compounds. During modeling, a crystal test set (TS_{CRY}, Supporting Information Table SI3) was compiled with more recently published co-crystallized complexes. TS_{CRY} was compiled with 22 non-covalent (**89-110**) and 25 covalent M^{pro} inhibitors (**111-135**) with various scaffolds (peptidomimetic, flavonoids and others), selected in order to maintain consistency with the bioactivity assay and measurement (IC_{50}). Moreover, 6 compounds were removed from the TS_{MOD} because they were in the meantime co-crystallized and only used in the TS_{CRY}. The final TS_{MOD} (Supporting Information Table SI2) and TS_{CRY} were constituted of 67 (**22-88**) and 47 (**89-135**) molecules and they were used to validate 3-D QSAR and COMBINE models.

Ligand-based Py-CoMFA models.

A full ligand-based approach to build 3-D QSAR models was investigated. The dataset molecules ligand-based alignment was achieved through the Py-ConfSearch and the Py-Align web apps suited in the web server 3d-qsar.com. Py-ConfSearch allows to accomplish a total of 11 conformational analysis through three software with corresponding energy minimization force fields: OpenBabel (UFF, GAFF, MMFF94, MMFF94s, Ghemical), RDKit (UFF, MMFF94, MMFF94s) and Ballon (EEM, MMFF94, SFKEEM). A useful option in the Py-Align module allows to perform a systematical combination of alignments using ShaEP (using both scoring functions: Similarity, and OnlyShape) and RDKit (4 alignment methods: Best Score, Tanimoto Distance, Protrude Distance, Lowest RMSD) on 16 query molecules from each conformational analysis (the most/least active, the heaviest, the longest, the most flexible/rigid, the least/most polar, with the highest/lowest MR, HD, LogP) in both the longest or the global minima conformation. Automatically, a preliminary 3-D QSAR model in Py-CoMFA was built for each so-obtained aligned dataset.

Structure-based alignment assessment.

A docking assessment was performed using Py-Docking module in 3d-qsar.com. The method involves the ability evaluation of 2 docking programs (Plants/Smina) and 6 different scoring functions (ChemPLP, PLP, PLP95 / Vina, Vinardo, AD4 scoring) to reproduce the binding pose of known co-crystallized compounds through 4 procedures: experimental conformation cross-docking (ECCD - Supporting Information Table SI6), random conformation cross-docking (RCCD - Supporting Information Table SI7), experimental conformation re-docking (ECRD - Supporting Information Table SI8) and random conformation re-docking (RCRD - Supporting Information Table SI9). Results analysis showed a global low docking accuracy (DA) [22], meaning that no scoring function was able to reproduce the experimental binding poses with a reasonable low error. Thus, it follows the impossibility to simulate a plausible binding pose for test set compounds by using a docking protocol. For this reason, the alternative flexible alignment approach was conceived.

Applicability domain definition.

The applicability domain (AD) of the model was defined by means of a *k*-nearest neighbors (*k*-NN) approach [23-25]. It was computed from the distribution of molecular fingerprint-derived similarity values between each TR compound and its *k* Nearest Neighbors (kNN, $k = 1$) in the training set. Fingerprints were provided as Morgan circular fingerprints [26] while similarities were measured using the Tanimoto index. The AD threshold (AD_T) was calculated as the average of the aforementioned similarity value distribution ($\overline{s}$) plus twice its standard deviation (σ) and returned a value of 0.4 (Equation 1),

$$AD_T = \overline{s} + 2\sigma \qquad \text{(Equation 1)}$$

Hence, if the similarity of a test set compound to its kNN in the training set exceeded the identified threshold, the prediction was considered unreliable and the compound removed from the analysis.

Table SI1. The 21 M^{pro} inhibitors SMILES structures collected from literature for the training set.

Mol ID	PDB ID	SMILES	IC_{50} (μM)
**1**	6XA4	CSCC[C@H](NC(=O)[C@H](CC(C)C)NC(=O)[C@H](CC(C)C)NC(C)=O)C=O	0.97 [9]
**2**	6WTT	CC(C)C[C@H](NC(=O)OCC1=CC=CC=C1)C(=O)N[C@@H](C[C@@H]1CCNC1=O)C(O)S(O)(=O)=O	0.030±0.008 [9]<br>0.62±0.08 [10]<br>0.15±0.03 [14]
**3**	6XHM	COC1=CC=CC2=C1C=C(N2)C(=O)N[C@@H](CC(C)C)C(=O)N[C@@H](CC1CCNC1=O)C(=O)CO	0.01 [3]
**4**	6XMK	O=C1NCC[C@H]1C[C@H](NC([C@@H](NC(OCC2CCC(F)(F)CC2)=O)CC(C)C)=O)C(O)C=O	0.48 [10]
**5**	6XBG	CC(C)C[C@H](NC(=O)OCC1=CC=CC=C1)C(=O)N[C@@H](CC1CCNC1=O)C(=O)C(=O)NC1CC1	0.05 [11]
**6**	6XBI	CC(C)C[C@H](NC(=O)OCC1=CC=CC=C1)C(=O)N[C@@H](CC(C)C)C(=O)N[C@@H](CC1CCNC1=O)C(=O)C(=O)NC1CC1	0.45 [11]
**7**	7JPZ	C=C(N[C@@H](C[C@@H]1CCNC1=O)C=O)[C@H](CC1=CC=CC=C1)NC(=O)OCC1=CC=CC=C1	0.10 [13]
**8**	7JQ0	CC(C)C[C@H](NC(=O)[C@@H](NC(=O)OCC1=CC=CC=C1)C(C)C)C(=C)NC(C[C@@H]1CCNC1=O)C=O	0.09 [13]
**9**	7JQ1	CC(C)[C@H](NC(=O)OCC1=CC=CC=C1)C(=O)N[C@@H](CC1=CC=CC=C1)C(=C)NC(C[C@@H]1CCNC1=O)C=O	0.02 [13]
**10**	7JQ2	CC(C)[C@H](NC(=O)OCC1=CC=CC=C1)C(=O)N[C@@H](CC1CCCCC1)C(=C)NC(C[C@@H]1CCNC1=O)C=O	0.03 [13]
**11**	7JQ3	CC(C)C[C@H](NC(=O)[C@@H](NC(=O)OCC1=CC=CC=C1)C(C)OC(C)(C)C)C(=C)NC(C[C@@H]1CCNC1=O)C=O	0.06 [13]
**12**	7JQ4	CC(OC(C)(C)C)[C@H](NC(=O)OCC1=CC=CC=C1)C(=O)N[C@@H](CC1=CC=CC=C1)C(=C)NC(C[C@@H]1CCNC1=O)C=O	0.05 [13]
**13**	7JQ5	CC(OC(C)(C)C)[C@H](NC(=O)OCC1=CC=CC=C1)C(=O)N[C@@H](CC1CCCCC1)C(=C)NC(C[C@@H]1CCNC1=O)C=O	0.11 [13]
**14**	6Y2F	CC(C)(C)OC(=O)NC1=CC=CN(C(CC2CC2)C(=O)NC(CC2CCNC2=O)C(=O)C(=O)NCC2=CC=CC=C2)C1=O	0.67 [12]
**15**	6LZE	O=CC(CC1CCNC1=O)NC(=O)C(CC1CCCCC1)NC(=O)C1=CC2=C(N1)C=CC=C2	0.05 [1]
**16**	6M0K	FC1=CC=CC(CC(NC(=O)C2=CC3=C(N2)C=CC=C3)C(=O)NC(CC2CCNC2=O)C=O)=C1	0.04 [1]
**17**	6XHM	COC1=CC=CC2=C1C=C(N2)C(=O)N[C@@H](CC(C)C)C(=O)N[C@@H](CC1CCNC1=O)C(=O)C1=NC2=CC=CC=C2S1	0.02 [2]
**18**	7BRP	CC(C)(C)NC(=O)N[C@H](C(=O)N1C[C@H]2[C@@H]([C@H]1C(=O)NC(CC1CCC1)C(=O)C(N)=O)C2(C)C)C(C)(C)C	3.1±0.4 [5]<br>4.13±0.61 [9]<br>8.0±1.5 [14]
**19**	7D1O	CCCC[C@H](NC(=O)[C@@H]1[C@@H]2[C@H](CN1C(=O)[C@@H](NC(=O)NC1(CS(=O)(=O)C(C)(C)C)CCCCC1)C(C)(C)C)C2(C)C)C(=O)C(=O)NC1CC1	5.1±0.9 [5]<br>5.73±0.67 [9]
**20**	7K6E	CCC[C@@H](C(=O)C(=O)NC1CC1)NC(=O)[C@@H]2[C@H]3CCC[C@H]3CN2C(=O)[C@H](C(C)(C)C)NC(=O)[C@H](C4CCCCC4)NC(=O)C5=NC=CN=C5	18.00 [5]
**21**	6XCH	CC(C)C[C@@H](C(=O)N[C@@H](CC(C)C)C(=O)N[C@@H](CCCN=C(N)N)C=O)NC(=O)C	92.00 [5]

Table SI2. The 67 M^{pro} inhibitors SMILES structures collected from literature for the TS_{MOD} test set.

Mol ID	SMILES	IC_{50} (μM)
**22**	CN(C)CCCSC1=CC=CC=C1NC(=O)C=CC2=CC=CC=C2	124.93 [4]
**23**	CCCCN1C(=O)C(=O)C2=C1C=CC(I)=C2	41.80 [27]
**24**	NC(=O)CC1=CC2=C(C=C1)N(CC1=CC=C3C=CC=CC3=C1)C(=O)C2=O	39.20 [27]
**25**	CC(C)C[C@H](NC(=O)[C@@H](NS(=O)(=O)C1=CC=C(F)C=C1)C(C)C)C=O	35.00 [9]
**26**	CC(C)[C@H](NC(=O)OCC1=CC=CC=C1)C(=O)N[C@@H](CC1=CC=CC=C1)C=O	25.00 [9]
**27**	NC(=O)C1=CC2=C(C=C1)N(CC1=CC=C3C=C(C=CC3=C1)C1=CC=CC=C1)C(=O)C2=O	24.90 [27]
**28**	CCCC(CC)SSC1=NC=CN1	21.39 [4]
**29**	CCCCN1C(=O)C(=O)C2=C1C=CC(=C2)C(N)=O	17.80 [27]
**30**	O=C(ON1C(=O)CCC1=O)C1=CC2=C(C=C1)N(CC1=CC=CC=C1)C(=O)C2=O	15.50 [27]
**31**	COC1=C(C)C2=C(C=C1)C(O[C@@H]1C[C@@H]3[C@@H](C1)C(=O)N(C)CCCC\C=C/[C@@H]1C[C@]1(N<br>C3=O)C(=O)NS(=O)(=O)C1CC1)=CC(=N2)C1=NC(=CS1)C(C)C	13.74 [9]
**32**	CCCC[C@H](NC(=O)[C@H](CC(C)C)NC(=O)OCC1=CC=CC=C1)C=O	10.69 [9]
**33**	CC[C@@H](C)[C@H](NC(=O)OCC1=CC=CC=C1)C(=O)N[C@@H](CCC(=O)OC(C)(C)C)C(=O)N[C@@H](C)<br>C(=O)C[C@@H](CC(C)C)C=O	10.38 [9]
**34**	CCCN1C(=O)C(=O)C2=C1C=CC(=C2)C(N)=O	10.20 [27]
**35**	CCCC[C@H](NC(=O)[C@H](CC(C)C)NC(=O)[C@H](CC(C)C)NC(C)=O)C=O	8.60 [9]
**36**	CCN(CC)C(=S)SSC(=S)N(CC)CC	7.00 [4]
**37**	CC(=O)C1OC(OC2=C(O)C(O)=C3C(=O)C=C(OC3=C2)C2=CC=CC=C2)C(O)C(O)C1O	6.41 [8]
**38**	OC1=CC=C(C=C1)C1=CC(=O)C2=C(O)C(O)=C(O)C=C2O1	5.80 [7]
**39**	CC(C)C[C@H](NC(=O)[C@H](CC(C)C)NC(=O)[C@H](CC(C)C)NC(=O)OCC1=CC=CC=C1)C=O	3.90 [9]
**40**	CCC[C@H](NC(=O)[C@H](CC(C)C)NC(=O)[C@H](CC(C)C)NC(=O)OCC1=CC=CC=C1)C=O	3.14 [9]
**41**	CC(C)(C)OC(=O)NC1=CC=CN(C(CC2CCCCC2)C(=O)NC(CC2CCNC2=O)C(=O)C(=O)NC2CC2)C1=O	2.39 [12]
**42**	CCCCCCNC(=O)N1C=C(C(=O)NC1=O)F	1.82 [4]
**43**	C1=CC=C(C=C1)CN2C(=O)N(SC2=O)C3=CC=CC4=CC=CC=C43	1.55 [4]
**44**	OC1=CC=C(C=C1O)C1=C(O)C(=O)C2=C(O)C(O)=C(O)C=C2O1	1.24 [7]
**45**	OC1C(OC2=CC(O)=CC(O)=C2C1=O)C1=CC(O)=C(O)C(O)=C1	1.20±0.09 [7]<br>1.14±0.03 [28]
**46**	O=C(NCC1)[C@@H]1C[C@@H](C=O)NC([C@H](CC(C)C)NC(OC2CCCCC2)=O)=O	0.82 [10]
**47**	O=CC(CC1CCNC1=O)NC(=O)C1C2CCCC2CN1C(=O)C1=CN=CC=C1	0.75 [21]
**48**	O=C1N(SC2=CC=CC=C12)C1=CC=CC=C1	0.67 [4]
**53**	O=CC(CC1CCNC1=O)NC(=O)C1C2CCCC2CN1C(=O)\C=C\C1=CC=CC=C1	0.53 [21]
**49**	CCC[C@H](NC(=O)[C@H](CC(C)C)NC(=O)OCC1=CC=CC=C1)C(=O)NCC1=CC=CC=N1	0.45 [9]
**50**	CC1(C)C2CN(C(C12)C(=O)NC(CC1CCNC1=O)C=O)C(=O)C1=CC2=C(C=CC=C2)C=C1	0.44 [21]
**51**	O=C(NCC1)[C@@H]1C[C@@H](C=O)NC([C@H](CC(C)C)NC(OC2CCC(F)(F)CC2)=O)=O	0.43 [10]
**52**	CN(C)C1=CC=C(CCC(=O)N2CC3CCCC3C2C(=O)NC(CC2CCNC2=O)C=O)C=C1	0.38 [21]
**53**	CN(C)C1=CC=C(\C=C\C(=O)N2CC3CCCC3C2C(=O)NC(CC2CCNC2=O)C=O)C=C1	0.38 [21]
**54**	CC1=CC(OC(=O)C2=CC=CC3=C2CCN3)=CN=C1	0.32 [29]
**55**	O=CC(CC1CCNC1=O)NC(=O)C1C2CCCC2CN1C(=O)C1=NN2C=CC=CC2=C1	0.30 [21]
**56**	O=C(NCC1)[C@@H]1C[C@@H](C=O)NC([C@H](CC(C)C)NC(OC2CCC(CCC)CC2)=O)=O	0.28 [10]
**57**	COC1=CC=C(\C=C\C(=O)N2CC3CCCC3C2C(=O)NC(CC2CCNC2=O)C=O)C(OC)=C1	0.20 [21]
**58**	O=C(NC(CC1CCCCC1)C(=O)NC(CC1CCNC1=O)C(=O)C(=O)NCC1=CC=CC=C1)\C=C\C1=CC=CC=C1	0.18 [12]
**59**	O=C(NCC1)[C@@H]1C[C@@H](C=O)NC([C@H](CC(C)C)NC(OC2CCC(CCCC)CC2)=O)=O	0.17 [10]
**60**	O=CC(CC1CCNC1=O)NC(=O)C1C2CCCC2CN1C(=O)C1=CC2=C(N1)C=CC=C2	0.15 [21]
**61**	FC1=CC(Cl)=CC=C1\C=C\C(=O)N[C@@H](CC1=CC=CC=C1)C(=C)N[C@@H](C[C@@H]1CCNC1=O)C=O	0.10 [30]

**62**	O=CC(CC1CCNC1=O)NC(=O)C1C2CCCC2CN1C(=O)COC1=CC=C2OCCOC2=C1	0.09 [21]
**63**	COC1=CC=C(OC(C)C(=O)N2CC3CCCC3C2C(=O)NC(CC2CCNC2=O)C=O)C=C1	0.07 [21]
**64**	CC(C)C[C@H](NC(=O)[C@@H](NC(=O)\C=C\C1=C(F)C=C(Cl)C=C1)C(C)C)C(=C)N[C@@H](C[C@@H]1CCNC1=O)C=O	0.06 [21]
**65**	NC(=O)C1=CC2=C(C=C1)N(CC1=CC3=CC=CC=C3S1)C(=O)C2=O	0.05 [21]
**66**	CC1(C)C2CN(C(C12)C(=O)NC(CC1CCNC1=O)C=O)C(=O)COC1=CC2=C(C=CC=C2)C=C1	0.05 [30]
**67**	CC1(C)C2CN(C(C12)C(=O)NC(CC1CCNC1=O)C=O)C(=O)COC1=CC(Br)=C(Cl)C=C1	0.05 [27]
**68**	NC(=O)C1=CC2=C(C=C1)N(CC1=CC=C3C=C(Br)C=CC3=C1)C(=O)C2=O	0.05 [21]
**69**	NC(=O)C1=CC2=C(C=C1)N(CC1=CC=C3C=CC=CC3=C1)C(=O)C2=O	0.05 [21]
**70**	CC1(C)C2CN(C(C12)C(=O)NC(CC1CCNC1=O)C=O)C(=O)COC1=CC=CC(=C1)C(F)(F)F	0.04 [27]
**71**	COC1=CC=C(OCC(=O)N2CC3C(C2C(=O)NC(CC2CCNC2=O)C=O)C3(C)C)C=C1F	0.04 [27]
**72**	COC1=CC=C(OCC(=O)N2CC3CCCC3C2C(=O)NC(CC2CCNC2=O)C=O)C=C1	0.04 [21]
**73**	FC1=C(OCC(=O)N2CC3CCCC3C2C(=O)NC(CC2CCNC2=O)C=O)C=CC(Cl)=C1	0.03 [21]
**74**	ClC1=CC=C(OCC(=O)N2CC3CCCC3C2C(=O)NC(CC2CCNC2=O)C=O)C=C1Cl	0.03 [21]
**75**	CC1=C(OCC(=O)N2CC3CCCC3C2C(=O)NC(CC2CCNC2=O)C=O)C=CC(Cl)=C1	0.02 [21]
**76**	CC1(C)C2CN(C(C12)C(=O)NC(CC1CCNC1=O)C=O)C(=O)COC1=CC=C(Cl)C=C1	0.02 [21]
**77**	CC1(C)C2CN(C(C12)C(=O)NC(CC1CCNC1=O)C=O)C(=O)COC1=CC(F)=CC(F)=C1	0.02 [21]
**78**	ClC1=CC(Cl)=C(OCC(=O)N2CC3CCCC3C2C(=O)NC(CC2CCNC2=O)C=O)C=C1	0.02 [21]
**79**	FC1=CC=C(CCC(=O)N2CC3CCCC3C2C(=O)NC(CC2CCNC2=O)C=O)C=C1F	0.02 [21]
**80**	CC1(C)C2CN(C(C12)C(=O)NC(CC1CCNC1=O)C=O)C(=O)COC1=CC=CC(F)=C1	0.02 [21]
**81**	CC1(C)C2CN(C(C12)C(=O)NC(CC1CCNC1=O)C=O)C(=O)COC1=CC=C(OC(F)(F)F)C=C1	0.02 [21]
**82**	COC1=CC=C(OCC(=O)N2CC3C(C2C(=O)NC(CC2CCNC2=O)C=O)C3(C)C)C=C1OC	0.01 [21]
**83**	CC1(C)C2CN(C(C12)C(=O)NC(CC1CCNC1=O)C=O)C(=O)COC1=CC=C(Br)C=C1Cl	0.01 [21]
**84**	CC1(C)C2CN(C(C12)C(=O)NC(CC1CCNC1=O)C=O)C(=O)COC1=CC=C(Cl)C=C1Cl	0.01 [21]
**85**	CC1(C)C2CN(C(C12)C(=O)NC(CC1CCNC1=O)C=O)C(=O)COC1=CC=C(F)C(F)=C1	0.01 [21]
**86**	CC1(C)C2CN(C(C12)C(=O)NC(CC1CCNC1=O)C=O)C(=O)COC1=CC(Cl)=C(Cl)C=C1	0.01 [21]
**87**	ClC1=CC=C(OCC(=O)N2CC3CCCC3C2C(=O)NC(CC2CCNC2=O)C=O)C=C1	0.01 [21]
**88**	O=CC(CC1CCNC1=O)NC(=O)C1C2CCCC2CN1C(=O)C1=CN=CC=C1	0.01 [21]

Table SI3. The 47 M^{pro} inhibitors SMILES structures collected from literature for the TS_{CRY} test set.

Mol ID	PDB ID	SMILES	IC$_{50}$ (μM)
**89**	7NT3	CCCN(C(C(=O)NCC1=CC2=C(OCO2)C=C1)C1=CC=CC(O)=C1)C(=O)C=C	200.00 [31]
**90**	7P51	ClC1=CN=C(NC(=O)C2CC(=O)C3=C2C=CC=C3)C=C1	54.00 [32]
**91**	7CA8	CC(=CCC(C1=CC(=O)C2=C(C=CC(=C2C1=O)O)O)O)C	15.75 [4]
**92**	7LME	O=C(CN1NNC2CCCCC12)N(CC1CCSC1)C1CCC(CC1)NC(=O)C1CC1	4.99 [33]
**93**	7L10	ClC1=CC(=CC(Cl)=C1)C1=CC(=CN(C2=CN=CC=C2)C1=O)C1=CC=CC=C1C#N	4.02 [34]
**94**	7L0D	CC(C)(C)NC(=O)C(N(C(=O)C1=CC=CO1)C1=CC=C(C=C1)C(C)(C)C)C1=CC=CN=C1	2.50 [35]
**95**	6M2N	OC1=C(O)C(O)=C2C(=O)C=C(OC2=C1)C1=CC=CC=C1	0.39±0.11 [7]<br>0.94±0.20 [8]
**96**	7M8X	COCCOC1=CC(Cl)=CC(=C1)C1=CC(=CN(C1=O)C1=CN=CC=C1)C1=CC=CC=C1C#N	0.47 [36]
**97**	7M90	ClC1=CC(=CC(OCCN2CCNC(=O)C2)=C1)C1=CC(=CN(C1=O)C1=CN=CC=C1)C1=CNC(=O)NC1=O	0.40 [36]
**98**	7M8Z	CC(C)(O)CCOC1=CC(Cl)=CC(=C1)C1=CC(=CN(C1=O)C1=CN=CC=C1)C1=CNC(=O)NC1=O	0.40 [36]
**99**	7L14	ClC1=CC(=CC(OCC2CC2)=C1)C1=CC(=CN(C1=O)C1=CN=CC=C1)C1=CC=CC=C1C#N	0.17 [34]
**100**	7L11	CCCOC1=CC(Cl)=CC(=C1)C1=CC(=CN(C1=O)C1=CN=CC=C1)C1=CC=CC=C1C#N	0.14 [34]
**101**	7L12	ClC1=CC(=CC(OCC2=CC=CC=C2)=C1)C1=CC(=CN(C1=O)C1=CN=CC=C1)C1=CNC(=O)NC1=O	0.13 [34]
**102**	7M8M	CCCOC1=CC(Cl)=CC(=C1)C1=CC(=CN(C1=O)C1=CN=CC=C1)C1=CNC(=O)NC1=O	0.12 [36]
**103**	7M8Y	ClC1=CC(=CC(OCCC2=CC=CC=C2)=C1)C1=CC(=CN(C1=O)C1=CN=CC=C1)C1=CNC(=O)NC1=O	0.11 [36]
**104**	7LMD	O=C(CN1NNC2CCCCC12)N(CC1CCSC1)C1CCC(CC1)-C1CN[NH]C1	0.11 [33]
**105**	7M8N	CC1=C(COC2=CC(CL)=CC(=C2)C2=CC(=CN(C2=O)C2=CN=CC=C2)C2=CNC(=O)NC2=O)C=CC=C1	0.10 [36]
**106**	7LMF	O=C(CN1NNC2CCCCC12)N(CC1CCSC1)C1CCC(CC1)-C1C[NH]CN1	0.06 [33]
**107**	7M8O	FC1=CC=CC(COC2=CC(Cl)=CC(=C2)C2=CC(=CN(C2=O)C2=CN=CC=C2)C2=CNC(=O)NC2=O)=C1	0.04 [36]
**108**	7M91	FC(F)(F)CCOC1=CC(Cl)=CC(=C1)C1=CC(=CN(C1=O)C1=CN=CC=C1)C1=CNC(=O)NC1=O	0.02 [36]
**109**	7M8P	FC1=C(OCC2=C(Cl)C=CC=C2)C=C(C=C1Cl)C1=CC(=CN(C1=O)C1=CN=CC=C1)C1=CNC(=O)NC1=O	0.02 [36]
**110**	7L13	ClC1=CC(=CC(OCC2=C(Cl)C=CC=C2)=C1)C1=CC(=CN(C1=O)C1=CN=CC=C1)C1=CNC(=O)NC1=O	0.02 [34]
**111**	7NT1	CCC(OC(=O)C=C)C(=O)NCCC1=CNC2=CC=CC=C12	139.10 [31]
**112**	7P35	CCOC(=O)\C=C\C(CC1CCNC1=O)NC(=O)C(CC(=O)[C@@H](NC(=O)C1=NOC(C)=C1)C(C)C)CC1=CC=C(F)C=C1	68.00 [37]
**113**	7NT2	COC1=C(OC)C(CNC(=O)C(OC(=O)C=C)C2=CC=C(C=C2)N(=O)=O)=CC=C1	9.39 [31]
**114**	7NUK	CCCN(CC(=O)NC1=CC=CC=C1OC)C(=O)CCl	3.96 [31]
**115**	7BE7	OC1=CC(O)=C2C(OC(=C(O)C2=O)C2=CC(O)=C(O)C(O)=C2)=C1	3.90 [9]
**116**	7AEH	O=C(NC(CC1=CC=CC=C1)C(=O)C(=O)NCC1=CC=CC=N1)C1CCC(=O)N1CC1=CC=CC=C1	3.00 [38]
**117**	7NTV	ClCC(=O)NC1=C(C=C(S1)C1=CC=CC=C1)C(=O)NC1CC1	2.36 [31]
**118**	7RC0	ClC1=CN=CC(OC(C2=CC=CC3=C2C=CN3)=O)=C1C	2.22 [39]
**119**	7B3E	OC1=CC2=C(C(O)=C1)C(=O)C(O)=C(O2)C1=CC(O)=C(O)C(O)=C1	0.63±0.01 [28]<br>2.86±0.23 [7]
**120**	7RBZ	ClC1=CC(OC(C2=CC=CC3=C2CCN3)=O)=CN=C1	0.32 [39]
**121**	7DPU	COC1=CC2=C(C(O)=C1)C(=O)C(O)=C(O2)C1=CC(O)=C(O)C(O)=C1	0.30 [28]
**122**	7LKW	O=C(N[C@@H](CC(C)C)C(N[C@@H](C[C@H]1C(NCC1)=O)C(O)S(=O)(O[Na])=O)=O)OC([C@H]2C[C@@H]3C[C@@H](C=CC3)C2)([2H])[2H]	0.30 [40]
**123**	7LKU	O=C(N[C@@H](CC(C)C)C(N[C@@H](C[C@H]1C(NCC1)=O)C(O)S(=O)(O[Na])=O)=O)OC([C@H]2C[C@@H]3C[C@@H](CCC3)C2)([2H])[2H]	0.29 [40]
**124**	7LKX	O=C(N[C@@H](CC(C)C)C(N[C@@H](C[C@H]1C(NCC1)=O)C(O)S(=O)(O[Na])=O)=O)OCC2C(C3)C=CC3C2	0.28 [40]
**125**	7LKV	O=C(NCC1)[C@@H]1C[C@@H](C(O)S(=O)(O[Na])=O)NC([C@H](CC(C)C)NC(OCC2C[C@@H]3C[C@H](C2)C=CC3)=O)=O	0.26 [40]
**126**	7DPV	COC1=CC2=C(C(O)=C1)C(=O)C(O)C(O2)C1=CC(O)=C(O)C(O)=C1	0.26 [28]

**127**	7LKR	O=C(NCC1)[C@@H]1C[C@@H](C=O)NC([C@H](CC(C)C)NC(OCC2C[C@@H]3C[C@H](C2)CCC3)=O)=O	0.18 [40]
**128**	7LKT	O=C(N[C@@H](CC(C)C)C(N[C@@H](C[C@H]1C(NCC1)=O)C([H])=O)=O)OCC23C[C@@H]4C[C@H](C3)C[C@H](C2)C4	0.18 [40]
**129**	7JW8	CCOC(=O)\C=C\C(CC1CCNC1=O)NC(=O)C(CC(C)C)NC(=O)C(NC(=O)OCC1=CC=CC=C1)C(C)OC(C)(C)C	0.15 [41]
**130**	7LKS	O=C(N[C@@H](CC(C)C)C(N[C@@H](C[C@H]1C(NCC1)=O)C([H])=O)=O)OCC2C(C3)CCC3C2	0.15 [40]
**131**	7JT0	ClC1=CN=CC(OC(=O)C2=CC=CS2)=C1	0.14 [41]
**132**	7LYH	[H]C(=O)C(CC1CCNC1=O)NC(=O)C1C2CCCC2CN1C(=O)OCC1=CC=CC=C1	0.05 [42]
**133**	7LYI	[H]C(=O)C(CC1CCNC1=O)NC(=O)C1C2C(CN1C(=O)OCC1=CC=CC=C1)C2(C)C	0.05 [42]
**134**	7AEG	CC(C)C(NC(=O)OCC1=CC=CC=C1)C(=O)NC(C)C(=O)NC(CC(O)=O)C(=O)COC(=O)C1=C(Cl)C=CC=C1Cl	0.03 [38]
**135**	7D3I	FC1=CC(CCC(=O)N2CC3CCCC3C2C(=O)NC(CC2CCNC2=O)C=O)=CC(F)=C1	0.01 [21]

Table SI4. TS_{MOD} compounds Tanimoto similarity index with the reference ligand associated to the PDB ID used for the fkcombu alignment.

Mol ID	Tanimoto Similarity Index	Reference PDB ID	Mol ID	Tanimoto Similarity Index	Reference PDB ID
**22**	0.20	7JKV	**56**	0.95	6XMK
**23**	0.34	6M0K	**57**	0.49	6XBI
**24**	0.36	6M0K	**58**	0.55	6XBG
**25**	0.35	6XA4	**59**	0.95	6XMK
**26**	0.84	7JQ1	**60**	0.98	6LZE
**27**	0.34	6M0K	**61**	0.55	7JQ1
**28**	0.11	6XA4	**62**	0.51	6XBI
**29**	0.36	6M0K	**63**	0.58	6XBI
**30**	0.32	6M0K	**64**	0.50	7JQ0
**31**	0.38	6XHM	**65**	0.34	6M0K
**32**	0.93	6WTT	**66**	0.59	6XBI
**33**	0.83	7JQ2	**67**	0.51	6XBI
**34**	0.33	6M0K	**68**	0.35	6M0K
**35**	0.73	6XCH	**69**	0.35	6M0K
**36**	0.14	6XCH	**70**	0.53	6XBI
**37**	0.15	7JQ2	**71**	0.55	6XBI
**38**	0.89	7JQ0	**72**	0.58	6XBI
**39**	0.91	6Y2F	**73**	0.52	6XBI
**40**	0.27	6XHM	**74**	0.54	6XBI
**41**	0.21	6M0K	**75**	0.55	6XBI
**42**	0.14	7JQ2	**76**	0.55	6XBI
**43**	0.24	7JQ4	**77**	0.53	6XBI
**44**	0.94	6XMK	**78**	0.61	7JPZ
**45**	0.65	6LZE	**79**	0.56	6XBI
**46**	0.25	6M0K	**80**	0.55	6XBI
**47**	0.15	7JQ2	**81**	0.55	6XBI
**48**	0.55	7JQ2	**82**	0.51	6XBI
**49**	0.63	7JQ0	**83**	0.53	6XBI
**50**	0.68	6LZE	**84**	0.55	6XBI
**51**	0.96	6XMK	**85**	0.54	6XBI
**52**	0.61	7JPZ	**86**	0.55	6XBI
**53**	0.51	7JQ2	**87**	0.62	7JPZ
**54**	0.25	6XHM	**88**	0.63	7JPZ
**55**	0.66	6LZE			

Table SI5. TS_{CRY} compounds Tanimoto similarity index with the most similar TR reference ligand.

Mol ID	Tanimoto Similarity Index	TR PDB ID	Mol ID	Tanimoto Similarity Index	TR PDB ID
**89**	0.35	6XHM	**113**	0.30	7JQ4
**90**	0.38	6M0K	**114**	0.33	6XBI
**91**	0.20	7JPZ	**115**	0.89	7JQ0
**92**	0.38	6XHM	**116**	0.69	6M0K
**93**	0.25	6Y2F	**117**	0.26	6LZE
**94**	0.40	6M0K	**118**	0.30	6XHM
**95**	0.15	7JQ2	**119**	0.15	7JQ2
**96**	0.26	6XHM	**120**	0.27	6XHM
**97**	0.32	6XHM	**121**	0.15	7JQ2
**98**	0.27	6XHM	**122**	0.54	6XMK
**99**	0.28	6XHM	**123**	0.58	6XMK
**100**	0.27	6XHM	**124**	0.52	6XMK
**101**	0.27	6XHM	**125**	0.54	6XMK
**102**	0.27	6XHM	**126**	0.24	7JQ4
**103**	0.26	6XHM	**127**	0.95	6XMK
**104**	0.34	6XHM	**128**	0.95	6XMK
**105**	0.26	6XHM	**129**	0.74	7JQ3
**106**	0.35	6XHM	**130**	0.94	6XMK
**107**	0.27	6Y2F	**131**	0.23	6XHM
**108**	0.28	6XHM	**132**	0.98	6WTT
**109**	0.27	6XHM	**133**	0.98	6WTT
**110**	0.26	6XHM	**134**	0.60	7JQ0
**111**	0.36	6M0K	**135**	0.62	7JPZ
**112**	0.36	6M0K			

Table SI6. Docking assessment by ECCD.

		SMINA									PLANTS								
		Vinardo			Vina			AD4 scoring			PLP			PLP95			ChemPLP		
**ID**	**PDB ID**	**Lock**	**RMSD**	**Energy**	**Lock**	**RMSD**	**Energy**	**Lock**	**RMSD**	**Energy**	**Lock**	**RMSD**	**Energy**	**Lock**	**RMSD**	**Energy**	**Lock**	**RMSD**	**Energy**
**1**	6XA4	6Y2F	7.95	-7.9	6Y2F	6.7	-7.3	6XMK	5.3	-42.2	7JKV	4.3	-87.0	6WTT	7.0	-144.6	7JKV	4.3	-87.0
**2**	6WTT	7JKV	2.12	-8.3	7D1O	3.1	-7.3	6XBG	7.2	-44.6	6XHM	2.0	-93.8	7JKV	1.4	-158.0	6XHM	2.1	-96.5
**3**	6XHM	7D1O	4.42	-8.9	6XCH	3.0	-7.9	6XBG	7.7	-46.1	6XHM	1.8	-100.3	6XHM	2.4	-170.7	7JKV	3.6	-100.4
**4**	6XMK	6WTT	9.72	-8.3	7D1O	4.7	-7.5	7JPZ	8.3	-45.5	7JQ4	7.8	-93.7	6XCH	7.3	-150.4	7JQ4	7.2	-100.4
**5**	6XBG	7K6E	4.26	-10.0	7D1O	5.9	-8.4	7D1O	9.5	-48.0	7JQ2	2.3	-102.4	7BRP	2.8	-174.8	7D1O	3.9	-104.9
**6**	6XBI	7JKV	2.9	-9.9	7K6E	10.0	-8.5	6LZE	4.5	-56.9	6WTT	3.3	-107.2	7D1O	4.8	-182.8	7D1O	4.8	-105.7
**7**	7JPZ	6WTT	9.17	-9.5	7K6E	5.5	-9.0	6XMK	8.0	-50.7	7JPZ	6.1	-105.5	7JQ1	2.8	-166.6	7K6E	6.3	-109.6
**8**	7JQ0	6WTT	6.19	-9.1	7D1O	8.1	-8.0	7JPZ	8.8	-52.7	7JQ4	6.6	-106.3	6WTT	3.4	-168.5	7JPZ	7.5	-104.7
**9**	7JQ1	6XCH	3.86	-9.6	7JPZ	8.9	-8.4	7JPZ	6.6	-54.8	7K6E	4.8	-111.8	7JKV	3.4	-177.5	7BRP	4.9	-114.0
**10**	7JQ2	7D1O	8.36	-10.1	7D1O	8.7	-9.6	7JPZ	9.2	-57.2	7K6E	6.6	-106.1	7JQ1	4.7	-172.4	7K6E	8.7	-104.2
**11**	7JQ3	7D1O	8.76	-11.0	6XBG	7.8	-11.6	7D1O	9.7	-58.0	7K6E	4.6	-99.4	6XA4	10.2	-169.0	7JQ2	2.8	-96.2
**12**	7JQ4	7K6E	11.38	-11.8	7K6E	10.1	-14.3	7K6E	9.9	-60.6	7K6E	4.4	-108.9	7K6E	10.0	-181.2	7D1O	4.6	-108.0
**13**	7JQ5	7D1O	4.61	-11.8	7D1O	10.6	-14.1	7K6E	10.4	-59.9	7JQ4	4.2	-102.0	6XMK	9.0	-172.1	6XBI	5.3	-102.3
**14**	6Y2F	6WTT	9.15	-9.3	7D1O	9.2	-8.5	7D1O	9.1	-54.9	7K6E	9.7	-109.4	7JQ2	3.5	-179.7	7JQ0	8.0	-106.7
**15**	6LZE	6XA4	1.59	-9.4	7JKV	7.1	-8.5	7JPZ	8.4	-49.9	6XBG	1.2	-98.0	7JKV	1.4	-167.0	7JPZ	3.2	-100.9
**16**	6M0K	6XCH	3.52	-9.5	7BRP	5.2	-8.9	6XMK	7.3	-50.8	7JKV	2.0	-104.7	6XHM	2.0	-173.2	7JKV	1.9	-118.1
**17**	7JKV	7D1O	8.83	-9.5	7K6E	9.3	-8.7	7K6E	8.2	-54.1	6XA4	4.5	-107.3	7JKV	2.4	-178.2	7D1O	3.7	-110.6
**18**	7BRP	7BRP	8.64	-10.3	7BRP	5.8	-10.8	6XBI	5.6	-47.8	7BRP	1.7	-96.6	7BRP	1.8	-159.9	7BRP	3.3	-103.7
**19**	7D1O	7D1O	4.58	-9.2	7D1O	8.7	-8.5	6XBG	10.2	-54.9	7K6E	10.3	-108.0	7K6E	4.6	-171.5	6XCH	7.6	-98.9
**20**	7K6E	7K6E	9.91	-9.8	7JPZ	12.9	-9.1	7K6E	10.2	-59.4	7D1O	9.1	-102.1	7BRP	5.1	-185.4	6XBG	11.3	-99.3
**21**	6XCH	6XMK	7.0	-7.8	7K6E	7.4	-6.8	7JPZ	5.8	-45.5	7JKV	2.5	-88.4	7K6E	8.0	-155.5	6XHM	2.9	-92.1
**DA (%)**			9.5			2.4			0.0			23.8			26.2			11.9	

ID: TR compound; PDB ID: PDB code associated to TR compound; Lock: Best-docked corresponding lock; RMSD: Best-docked Root-Mean-Square Deviation; Energy: Best-docked energy value; DA: Docking Accuracy.

Table SI7. Docking assessment by RCCD.

		SMINA									PLANTS								
		Vinardo			Vina			AD4 scoring			PLP			PLP95			ChemPLP		
ID	PDB ID	Lock	RMSD	Energy	Lock	RMSD	Energy	Lock	RMSD	Energy	Lock	RMSD	Energy	Lock	RMSD	Energy	Lock	RMSD	Energy
1	6XA4	7D1O	8.2	-8.5	7BRP	4.5	-7.3	6XMK	7.3	-42.4	7JKV	6.5	-86.2	6WTT	6.0	-146.1	6WTT	6.9	-92.4
2	6WTT	6XHM	2.6	-8.0	7D1O	3.5	-7.3	6XBG	7.2	-44.6	7K6E	8.1	-93.3	7JKV	1.7	-161.4	6WTT	3.1	-98.3
3	6XHM	7JPZ	8.6	-8.5	6XBG	2.1	-8.0	7JPZ	8.5	-50.5	6XHM	2.4	-100.5	7JQ0	3.1	-162.1	6XHM	2.3	-103.5
4	6XMK	7D1O	8.0	-8.6	7BRP	7.4	-7.3	6XMK	7.9	-45.3	7JPZ	6.4	-92.6	7JQ2	7.5	-145.5	6XCH	7.9	-95.0
5	6XBG	7K6E	4.4	-8.9	7D1O	5.6	-8.9	7JPZ	7.0	-49.0	7D1O	3.5	-103.0	7D1O	4.0	-173.0	7K6E	3.2	-100.0
6	6XBI	7D1O	10.3	-9.7	7D1O	10.2	-8.3	7K6E	4.1	-55.4	7JQ4	3.4	-107.4	7D1O	10.5	-180.1	7K6E	2.7	-110.6
7	7JPZ	7JPZ	1.8	-10.0	7BRP	23.6	-9.5	6XMK	6.5	-50.4	6WTT	7.4	-104.7	6XA4	7.0	-167.7	7K6E	8.2	-110.4
8	7JQ0	6XMK	5.5	-8.4	7D1O	3.3	-8.2	7JPZ	8.8	-54.0	6WTT	3.5	-105.4	6XHM	3.4	-167.8	7K6E	11.3	-111.1
9	7JQ1	7K6E	8.7	-10.0	7K6E	8.7	-8.4	7JPZ	8.9	-55.2	7K6E	7.2	-115.3	7D1O	8.7	-179.0	7K6E	3.7	-120.9
10	7JQ2	7D1O	3.0	-10.0	7D1O	8.8	-8.4	7JPZ	9.3	-55.7	7JQ4	5.4	-105.3	7D1O	3.3	-167.2	7K6E	4.2	-101.7
11	7JQ3	7JPZ	9.0	-9.9	7K6E	12.3	-12.1	7K6E	9.9	-58.8	7D1O	3.9	-100.5	7D1O	4.3	-169.4	7JPZ	10.3	-96.8
12	7JQ4	7BRP	9.2	-11.2	7K6E	10.2	-13.4	6XMK	7.2	-60.0	7D1O	8.6	-109.5	7JQ0	8.5	-178.9	7D1O	8.0	-106.4
13	7JQ5	7D1O	3.9	-11.6	7K6E	11.1	-12.9	7BRP	10.4	-60.3	7D1O	9.5	-102.3	6XMK	4.5	-181.5	7JQ4	3.2	-96.8
14	6Y2F	7D1O	4.3	-10.2	6LZE	8.1	-8.2	6XBG	8.6	-54.6	7K6E	9.7	-103.5	6XHM	8.5	-180.7	7K6E	9.5	-111.1
15	6LZE	7JQ2	6.3	-9.3	6XBG	1.6	-8.5	7JPZ	8.4	-49.5	7JKV	1.3	-101.2	7JPZ	1.8	-155.7	7JKV	1.4	-112.0
16	6M0K	6XBG	1.9	-10.1	7K6E	2.9	-8.5	7D1O	7.8	-47.9	7JKV	2.0	-105.8	7JKV	2.0	-177.1	7JKV	1.9	-117.4
17	7JKV	6Y2F	6.4	-9.3	7JPZ	8.2	-8.5	6XMK	8.5	-55.8	6WTT	10.0	-106.2	7JKV	2.6	-177.2	7K6E	10.5	-107.8
18	7BRP	7JQ4	3.8	-9.4	6M0K	9.7	-8.4	6XHM	7.7	-49.0	7JQ2	6.8	-97.7	7D1O	3.0	-152.2	6XCH	8.3	-94.0
19	7D1O	7K6E	9.4	-8.8	7K6E	5.5	-9.7	7D1O	9.6	-56.0	7K6E	5.1	-103.1	7K6E	5.0	-178.7	7JQ4	9.2	-87.8
20	7K6E	7K6E	5.6	-10.0	7K6E	10.3	-8.3	7D1O	10.2	-55.0	6LZE	10.0	-98.5	7D1O	5.6	-190.1	6Y2F	6.0	-106.2
21	6XCH	7K6E	7.4	-7.5	7K6E	7.4	-6.7	7JPZ	5.9	-44.6	6XMK	5.7	-87.3	6XMK	5.7	-158.0	6XMK	6.8	-89.7
DA (%)		14.3			9.5			0.0			11.9			14.3			14.3		

ID: TR compound; PDB ID: PDB code associated to TR compound; Lock: Best-docked corresponding lock; RMSD: Best-docked Root-Mean-Square Deviation; Energy: Best-docked energy value; DA: Docking Accuracy.

Table SI8. Docking assessment by ECRD.

		SMINA						PLANTS					
		Vina		Vinardo		AD4 scoring		PLP		PLP95		ChemPLP	
**ID**	**PDB ID**	**RMSD**	**Energy**	**RMSD**	**Energy**	**RMSD**	**Energy**	**RMSD**	**Energy**	**RMSD**	**Energy**	**RMSD**	**Energy**
**1**	6XA4	23.7	-6.7	24.1	-7.1	6.0	-37.7	8.0	-77.1	5.3	-128.1	5.4	-79.3
**2**	6WTT	5.0	-6.8	4.9	-7.3	8.0	-40.9	2.5	-88.7	3.1	-139.9	2.4	-87.0
**3**	6XHM	3.4	-7.2	3.5	-7.9	6.4	-42.0	1.8	-100.3	2.4	-170.7	1.5	-99.2
**4**	6XMK	7.5	-6.9	5.3	-7.4	8.2	-44.3	8.5	-85.3	6.5	-137.8	7.7	-80.7
**5**	6XBG	3.7	-7.6	4.5	-7.8	4.5	-46.0	6.6	-86.0	4.9	-166.3	2.6	-98.0
**6**	6XBI	4.5	-7.8	4.1	-8.1	7.7	-50.8	4.5	-100.6	1.8	-167.0	5.0	-98.0
**7**	7JPZ	7.2	-7.9	6.3	-9.4	7.5	-42.8	6.1	-105.5	6.1	-159.1	8.1	-105.7
**8**	7JQ0	9.3	-7.8	22.5	-6.9	4.9	-40.7	7.2	-98.1	6.6	-160.3	5.3	-95.7
**9**	7JQ1	9.2	-7.4	8.4	-8.0	9.0	-45.9	3.5	-104.8	6.5	-176.0	9.2	-98.8
**10**	7JQ2	6.0	-8.1	8.2	-8.7	7.5	-48.4	15.2	-67.4	6.3	-157.8	5.9	-100.0
**11**	7JQ3	8.7	-10.9	16.6	-9.9	3.9	-49.4	8.0	-87.7	2.4	-149.5	7.3	-87.9
**12**	7JQ4	7.1	-10.7	12.5	-10.1	10.0	-56.9	5.0	-102.3	7.0	-163.9	13.7	-97.1
**13**	7JQ5	9.2	-10.9	3.9	-9.8	7.8	-52.0	8.0	-42.4	9.9	-105.1	10.1	-41.3
**14**	6Y2F	5.4	-7.7	6.1	-7.8	8.3	-54.0	9.3	-103.7	9.6	-170.9	9.8	-98.0
**15**	6LZE	8.3	-7.8	8.1	-8.1	7.1	-45.5	7.5	-69.6	7.5	-142.5	7.2	-91.5
**16**	6M0K	7.0	-7.9	7.6	-8.5	5.8	-43.5	1.8	-100.6	8.0	-152.4	6.8	-96.6
**17**	7JKV	5.5	-7.9	19.1	-6.7	5.7	-51.6	10.1	-88.1	2.4	-178.2	10.3	-98.5
**18**	7BRP	5.8	-10.8	8.6	-10.3	8.1	-41.4	1.7	-96.6	1.8	-159.9	3.3	-103.7
**19**	7D1O	8.7	-8.5	4.6	-9.2	4.4	-53.5	10.0	-90.5	4.6	-107.0	6.9	11.6
**20**	7K6E	10.2	-8.4	9.9	-9.8	10.2	-59.4	9.1	-101.1	8.6	-92.7	9.7	-58.5
**21**	6XCH	6.5	-5.9	5.1	-6.2	5.8	-40.8	7.5	-80.6	6.6	-144.5	6.3	-82.0
**DA (%)**		0.0		0.0		0.0		16.7		16.7		9.5	

ID: TR compound; PDB ID: PDB code associated to TR compound; RMSD: Best-docked Root-Mean-Square Deviation; Energy: Best-docked energy value; DA: Docking Accuracy.

Table SI9. Docking assessment by RCRD.

		SMINA						PLANTS					
		Vina		Vinardo		AD4 scoring		PLP		PLP95		ChemPLP	
**ID**	**PDB ID**	**RMSD**	**Energy**	**RMSD**	**Energy**	**RMSD**	**Energy**	**RMSD**	**Energy**	**RMSD**	**Energy**	**RMSD**	**Energy**
**1**	6XA4	23.8	-6.7	24.0	-7.3	6.0	-37.8	6.5	-82.8	5.3	-136.8	5.8	-78.2
**2**	6WTT	2.5	-7.0	3.2	-7.0	7.4	-40.9	1.8	-90.8	1.9	-145.7	3.1	-98.3
**3**	6XHM	6.0	-7.2	3.5	-8.2	7.8	-43.3	2.4	-100.5	6.5	-156.2	2.3	-103.5
**4**	6XMK	7.3	-6.8	1.6	-8.4	7.9	-45.3	7.2	-79.0	8.2	-127.0	6.7	-94.5
**5**	6XBG	9.3	-7.6	8.5	-7.6	7.4	-43.0	8.5	-91.6	6.5	-148.9	5.0	-92.1
**6**	6XBI	10.1	-7.8	5.3	-8.6	6.0	-47.6	9.9	-100.1	6.2	-174.0	6.1	-94.2
**7**	7JPZ	7.3	-9.2	1.8	-10.0	7.1	-44.9	6.2	-102.6	8.2	-156.1	3.7	-99.6
**8**	7JQ0	10.0	-7.4	23.4	-7.5	8.3	-41.8	7.7	-90.7	2.4	-157.2	10.6	-91.4
**9**	7JQ1	8.4	-7.5	3.2	-8.4	8.6	-52.9	3.0	-101.6	8.9	-165.8	6.2	-105.0
**10**	7JQ2	6.2	-8.0	7.8	-9.0	8.1	-45.2	4.5	-104.2	5.8	-107.6	2.7	-96.2
**11**	7JQ3	8.1	-10.7	7.1	-9.6	6.4	-48.9	3.8	-92.2	8.0	-152.6	4.9	-89.2
**12**	7JQ4	8.9	-11.2	5.8	-10.1	11.5	-52.8	7.7	-103.8	9.1	-178.5	7.2	-96.1
**13**	7JQ5	9.2	-11.8	7.5	-10.0	10.9	-49.6	7.1	-36.1	2.6	-92.3	15.4	-48.0
**14**	6Y2F	4.7	-7.7	6.6	-7.1	8.1	-43.7	9.4	-98.1	9.5	-166.3	9.2	-100.8
**15**	6LZE	8.3	-7.5	7.7	-8.0	7.2	-43.4	5.9	-85.3	5.7	-152.3	2.0	-94.0
**16**	6M0K	7.9	-8.1	7.0	-6.8	5.5	-43.5	7.4	-94.2	1.8	-150.1	6.0	-97.1
**17**	7JKV	5.5	-8.2	8.1	-7.9	7.2	-49.2	2.3	-105.9	2.6	-177.2	8.7	-93.1
**18**	7BRP	8.1	-7.2	7.7	-8.2	5.3	-40.4	2.0	-96.3	3.2	-150.4	8.1	-84.5
**19**	7D1O	5.0	-9.0	8.1	-7.0	9.6	-56.0	6.0	-95.1	1.4	-151.3	5.0	-67.7
**20**	7K6E	10.3	-8.3	5.6	-10.0	6.2	-52.7	5.2	-97.6	5.1	-189.8	7.2	-99.2
**21**	6XCH	3.3	-5.5	14.6	-6.0	5.5	-41.0	6.2	-81.1	6.6	-138.1	6.0	-82.7
**DA (%)**		2.4		9.5		0.0		16.7		21.4		7.1	

ID: TR compound; PDB ID: PDB code associated to TR compound; RMSD: Best-docked Root-Mean-Square Deviation; Energy: Best-docked energy value; DA: Docking Accuracy.

Table SI10. Default and Variable Pre-treatment Optimization (VPO) parameters.

Parameters	Description	Type/Range/Step (steps)	Steps	Default
Probe types	SYBYL atom types.	Al. Br. C.1. C.2. C.3. C.1.H1. C.2.H1. C.2.H2. C.3.H1. C.3.H2. C.3.H3. C.ar. C.ar.H1. C.cat. Ca. Cl. F. H. H.spc. H.t3p. I. K. Li. N.1. N.2. N.3. N.4. N.am. N.ar. N.pl3. Na. O.2. O.3. O.co2. O.t3p. P.3. S.2. S.3. S.o. S.o2. Si	42	C.3
Grid spacing (Å)	Distance among grid intersections (atom probes).	1.0/3.0/0.1	30	2.0
Grid extension (Å)	Extension of the grid embracing the aligned training set molecules.	1.0/10.0/0.1	100	5.0
Dielectric constant	Value used for the calculation of the electrostatic molecular interactions fields with the Coulomb function (ELE)	0/80/1	80	8
Min/max cut-off energy (kcal/mol)	Maximum energy value. If the probe atom detects a greater energy. the steric interaction value is set on the defined value. while the electrostatic interaction to the mean of the other revealed values at the same location.	5/50/1	45	30
Minimum sigma	Minimum standard deviation value. Variables having a standard deviation below this value are removed.	0.05/1.00/0.05	20	0.05

Table SI11. Preliminary Py-CoMFA models with the default settings (probe type = C.3. grid spacing = 2.0 Å. grid extension = 5 Å. dielectric constant = 8. min/max cut-off energy value = 30 kcal/mol and minimum sigma = 0.05, see Table SI10). The cross-validation method used was the LOO. In yellow are highlighted the optimal models.

	MIFs											
**PC**	**STE**				**ELE**				**BOTH**			
	***r*²**	**SDEC**	***q*²**	**SDEP**	***r*²**	**SDEC**	***q*²**	**SDEP**	***r*²**	**SDEC**	***q*²**	**SDEP**
1	0.75	0.28	0.50	0.56	0.37	0.70	-0.07	1.19	0.47	0.59	0.09	1.00
2	0.90	0.11	0.58	0.47	0.81	0.21	0.28	0.80	0.92	0.09	0.63	0.41
3	0.96	0.05	0.59	0.46	0.92	0.09	0.49	0.57	0.97	0.03	0.66	0.38
4	0.99	0.01	0.57	0.48	0.95	0.05	0.53	0.52	0.99	0.01	0.63	0.41
5	1.00	0.00	0.55	0.50	0.98	0.03	0.54	0.51	1.00	0.00	0.64	0.40
6	1.00	0.00	0.55	0.50	0.99	0.02	0.52	0.53	1.00	0.00	0.65	0.39

Table SI12. Py-ComBinE models' statistical results

**Model**	**Fields**	r^2**(PC)**	q^2_{LOO}	r^2**(PC)**	q^2_{LSO}
**SB1**	STE	0.81(2)	0.56	0.81(2)	0.59
**SB2**	ELE	0.37(1)	-0.17	0.37(1)	0.31
**SB3**	DRY	0.70(2)	0.32	0.95(7)	0.60
**SB4**	HB	0.76(2)	0.48	0.76(2)	0.59
**SB5**	STE.ELE	0.90(3)	0.24	0.86(2)	0.42
**SB6**	STE.DRY	0.78(2)	0.49	0.78(2)	0.55
**SB7**	STE.HB	0.91(3)	0.69	0.91(3)	0.69
**SB8**	ELE.DRY	0.89(3)	0.06	0.40(1)	0.30
**SB9**	DRY.HB	0.88(3)	0.56	0.82(2)	0.58
**SB10**	ELE.HB	0.83(2)	0.16	0.43(1)	0.32
**SB11**	STE.ELE.DRY	0.86(2)	0.17	0.86(2)	0.46
**SB12**	STE.DRY.HB	0.91(3)	0.64	0.86(2)	0.64
**SB13**	ELE.DRY.HB	0.91(3)	0.18	0.85(2)	0.40
**SB14**	STE.ELE.HB	0.93(3)	0.35	0.87(2)	0.46
**SB15**	STE.ELE.DRY.HB	0.92(3)	0.38	0.87(2)	0.46

	AAC plot	Structure	IC_{50} (μM)
**A**	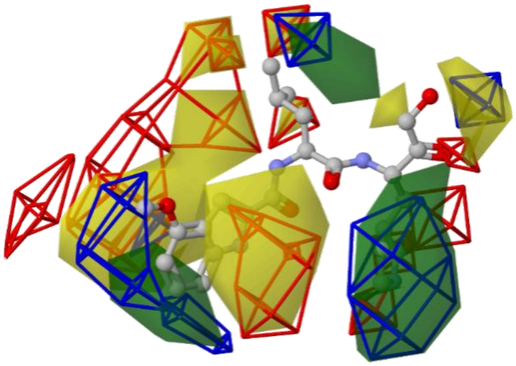	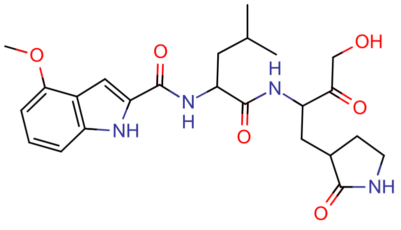 **3**	0.01
**B**	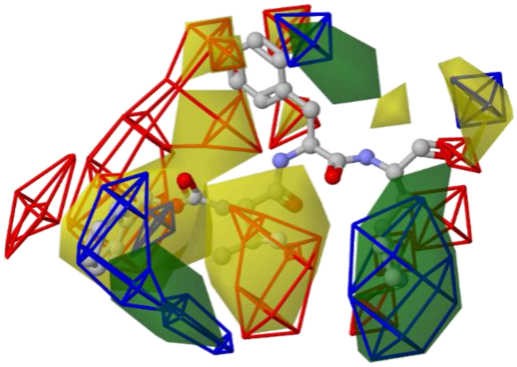	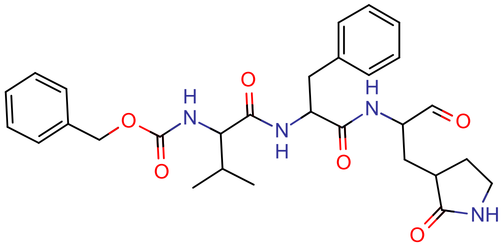 **9**	0.02
**C**	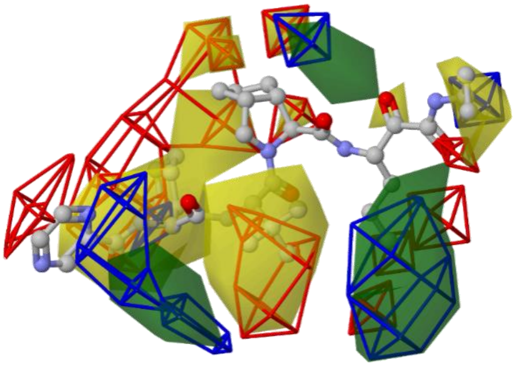	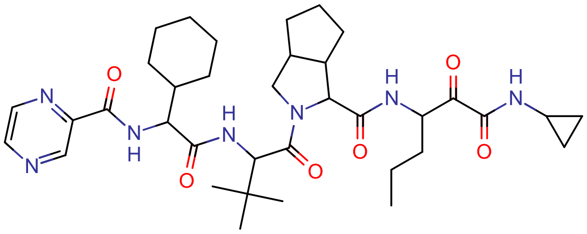 **20**	18.00
**D**	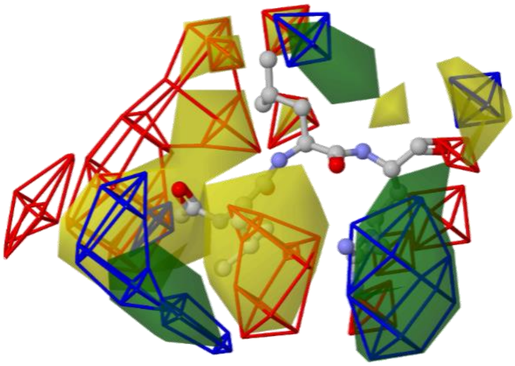	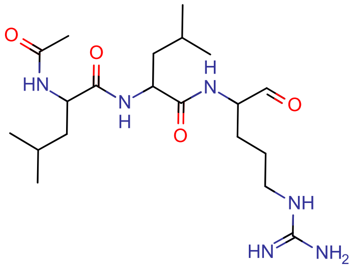 **21**	92.00

Figure SI1. Average Activity contribution (AAC) contour plots containing the 2 most active TR compounds 3 (**A**) and 9 (**B**) and the 2 less active TR compounds 20 (**C**) and 21 (**D**). Contour levels: 70% (steric: positive green. negative yellow; electrostatic: positive blue. negative red). Hydrogen atoms are omitted for the sake of clarity. Compounds are reported with the same orientation of compound 4 in Figure 2 and 5 (P4 – P1’). The plots were generated directly within the 3d-qsar.com portal.

ID	AC plot	Structure	IC_{50} (µM)
**1**	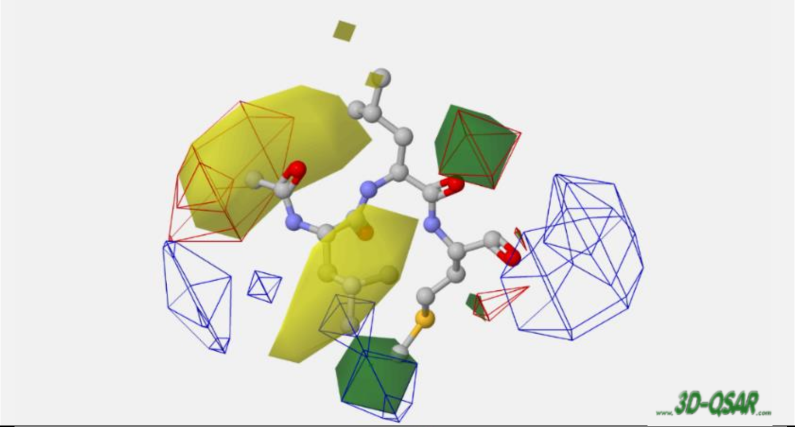	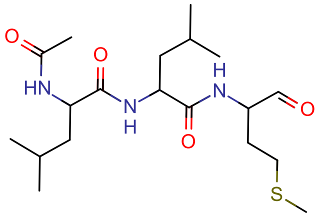	0.97
**2**	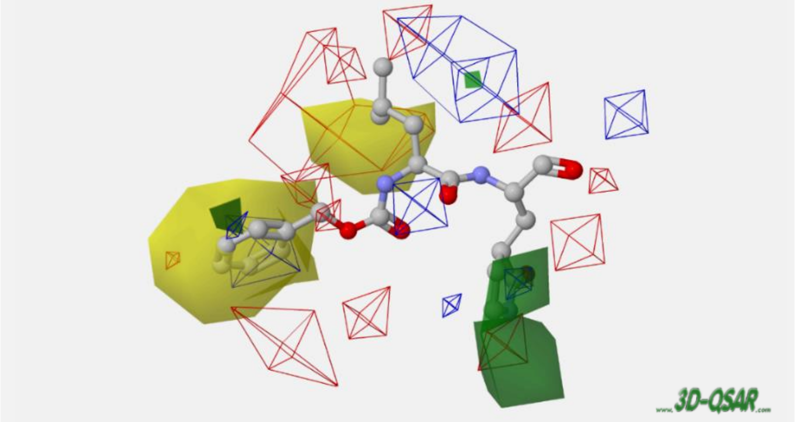	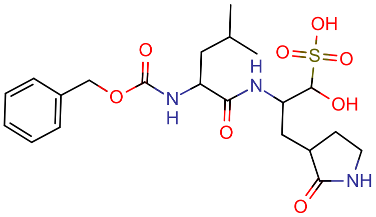	0.03<br>0.62<br>0.15
**3**	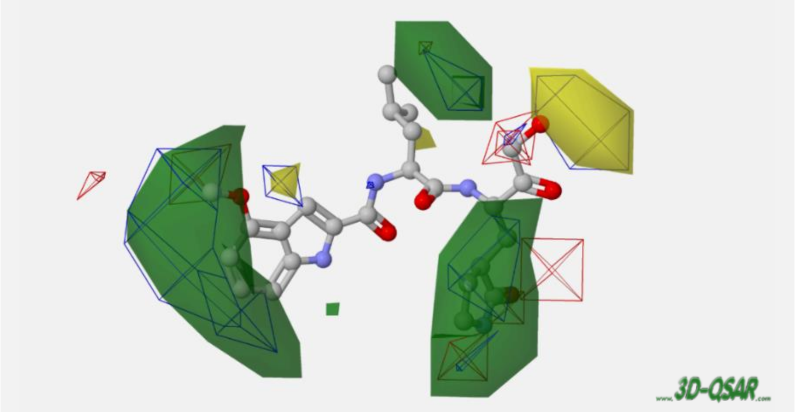	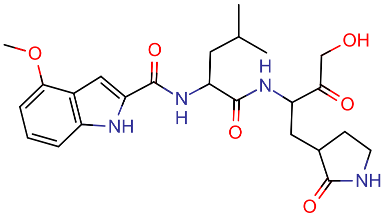	0.01
**4**	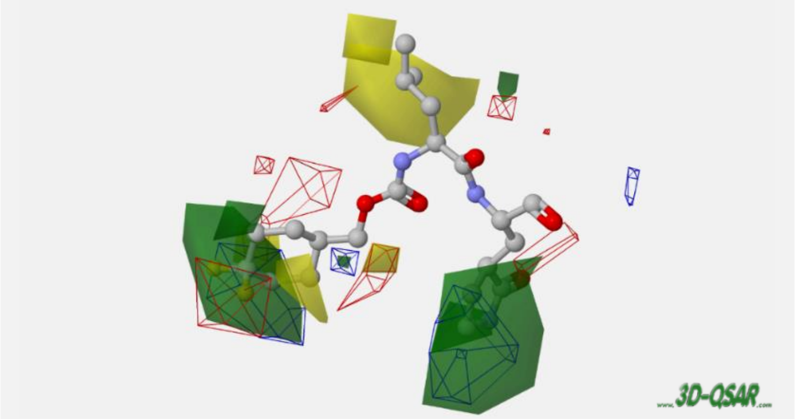	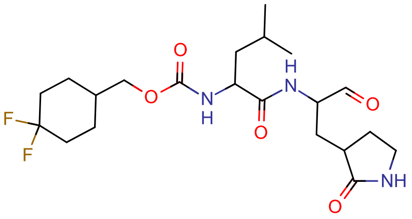	0.48

**5**	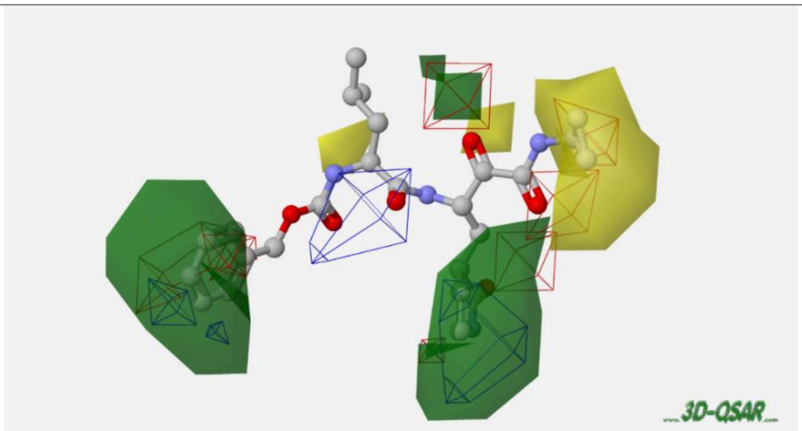	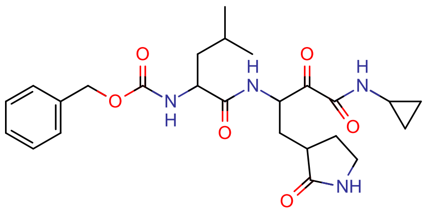	0.05
**6**	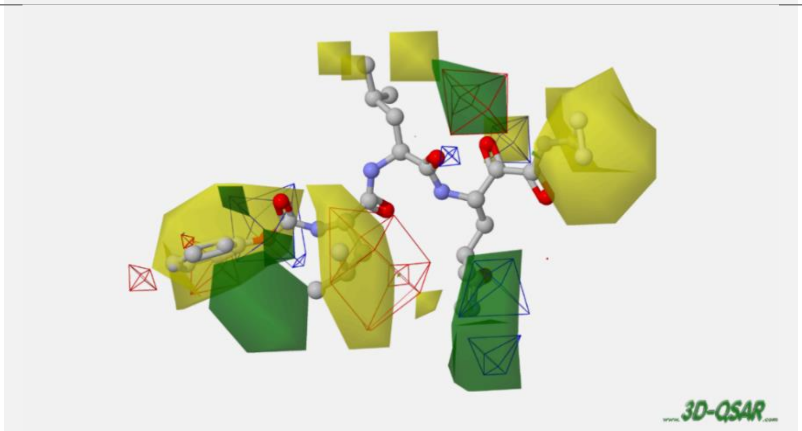	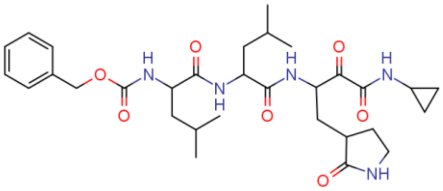	0.45
**7**	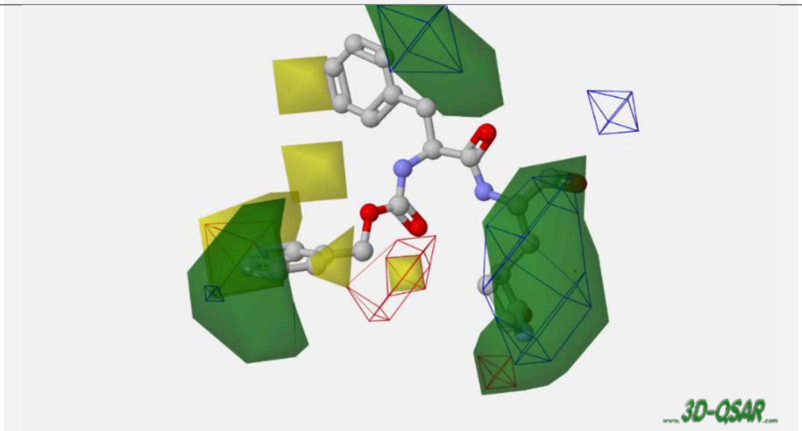	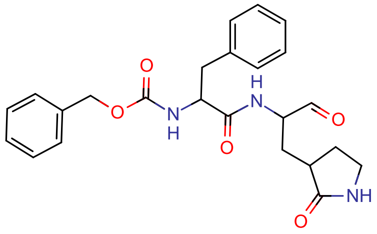	0.10
**8**	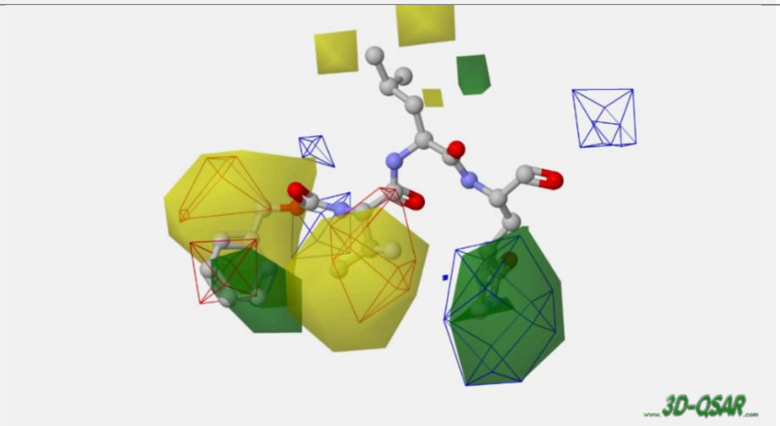	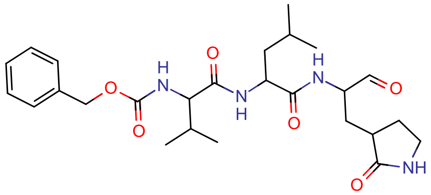	0.09

**9**	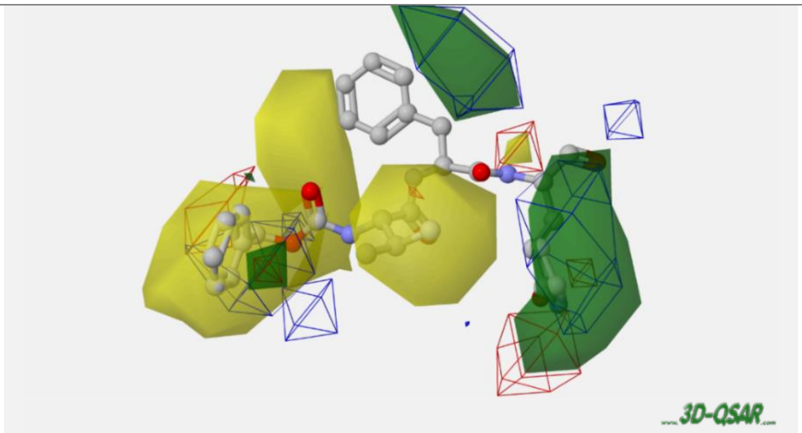	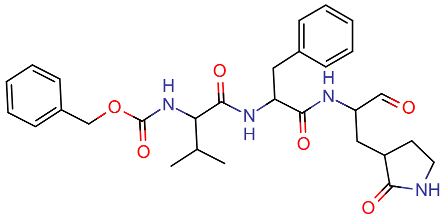	0.02
**10**	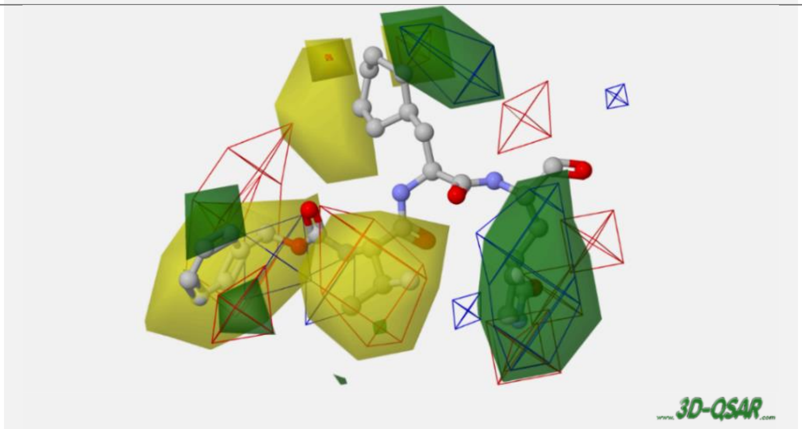	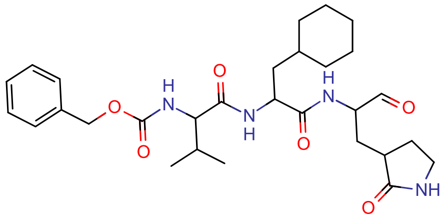	0.03
**11**	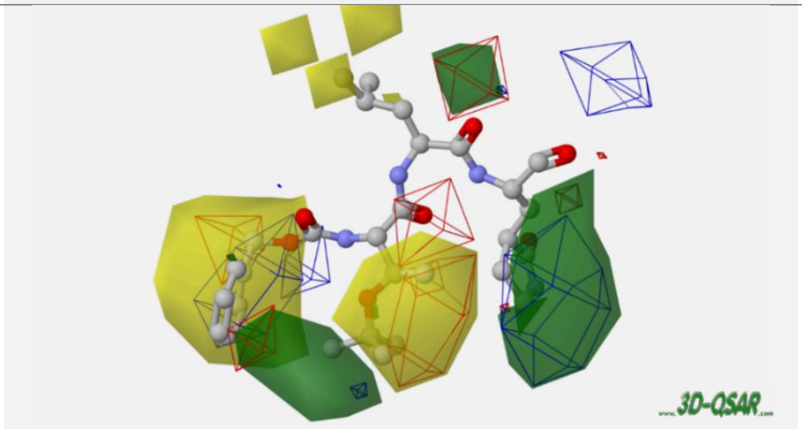	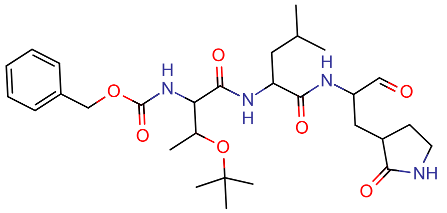	0.06
**12**	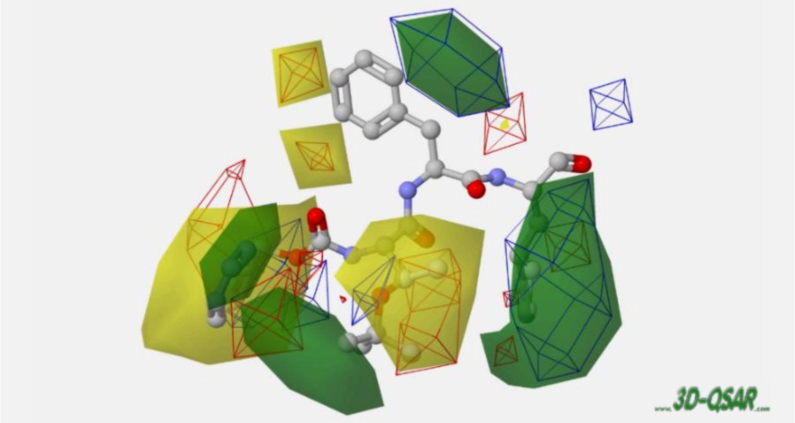	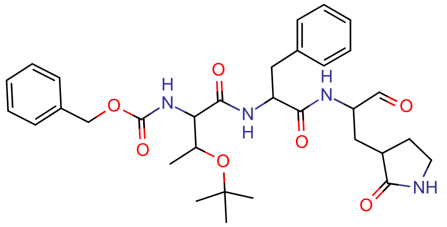	0.05

**13**	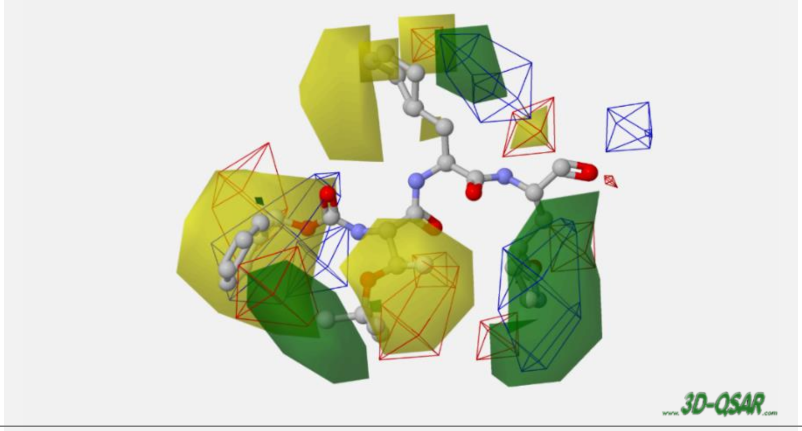	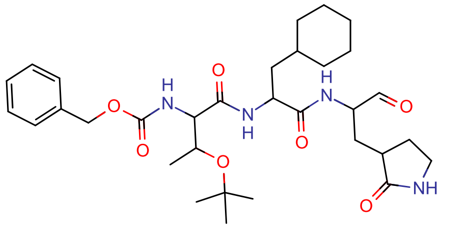	0.11
**14**	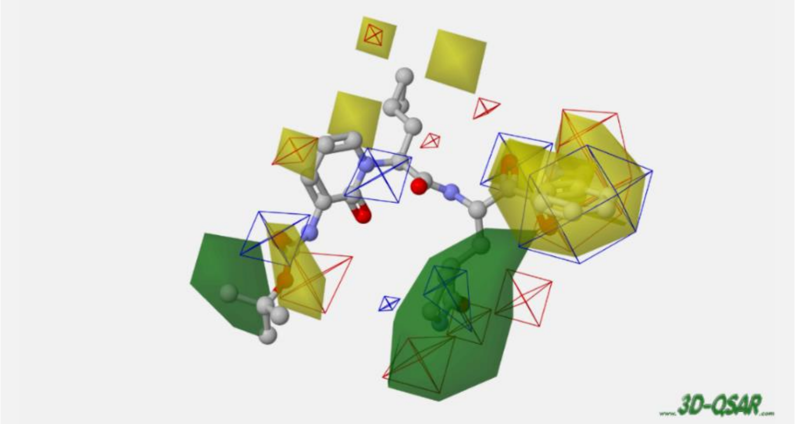	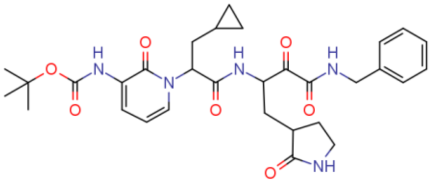	0.67
**15**	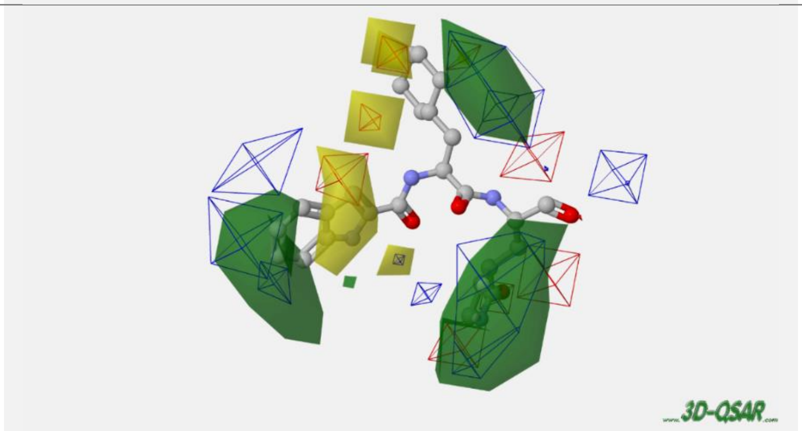	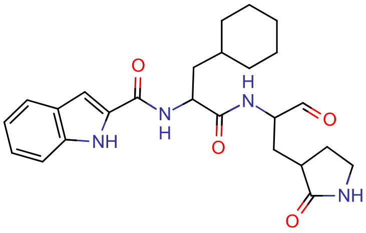	0.05
**16**	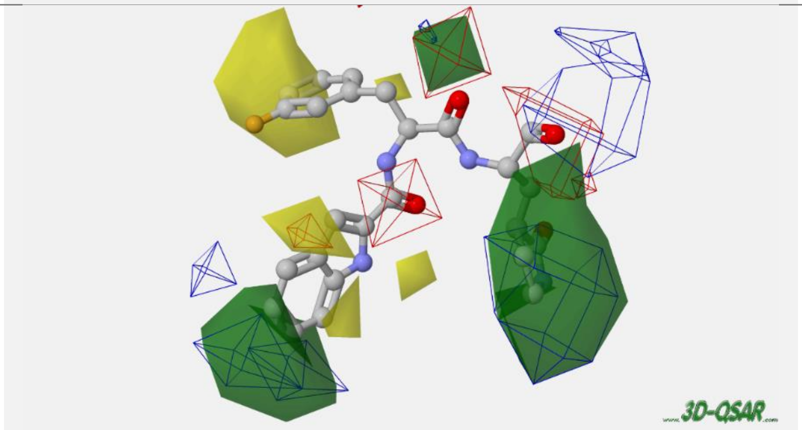	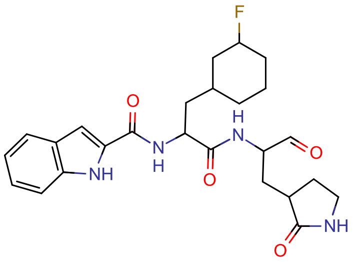	0.04

**17**	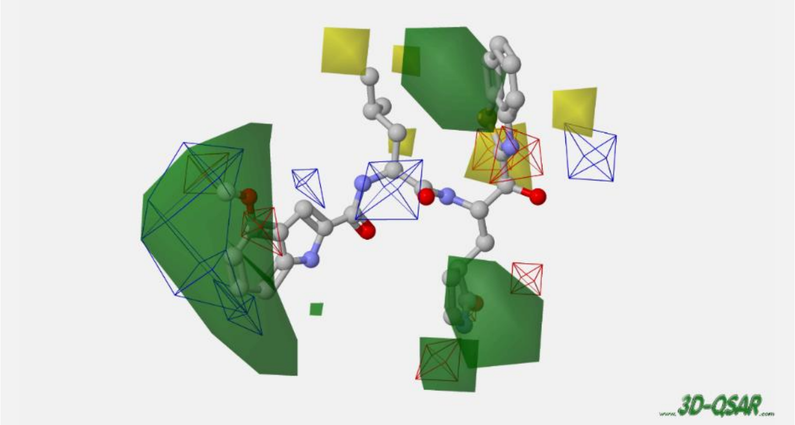	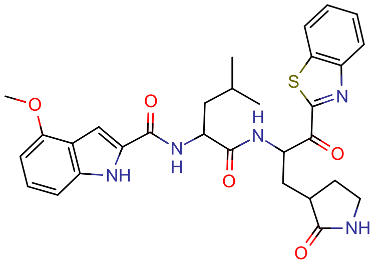	0.02
**18**	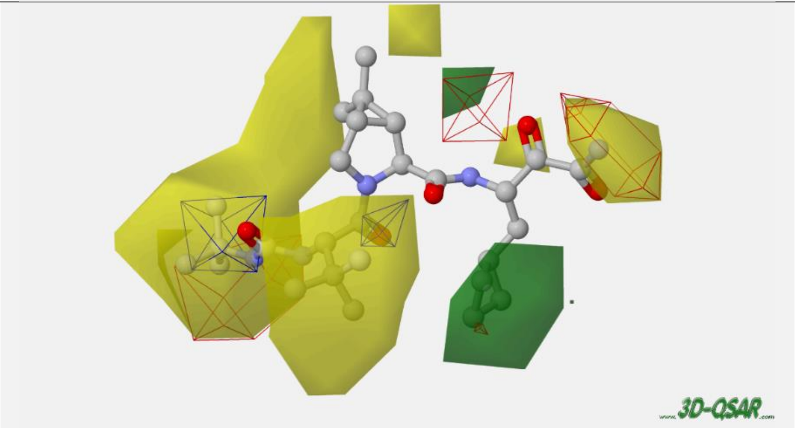	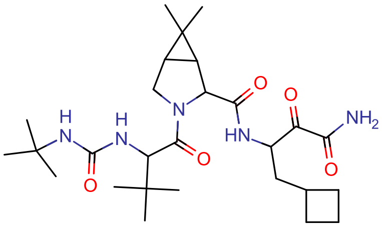	3.1<br>4.13<br>8.0
**19**	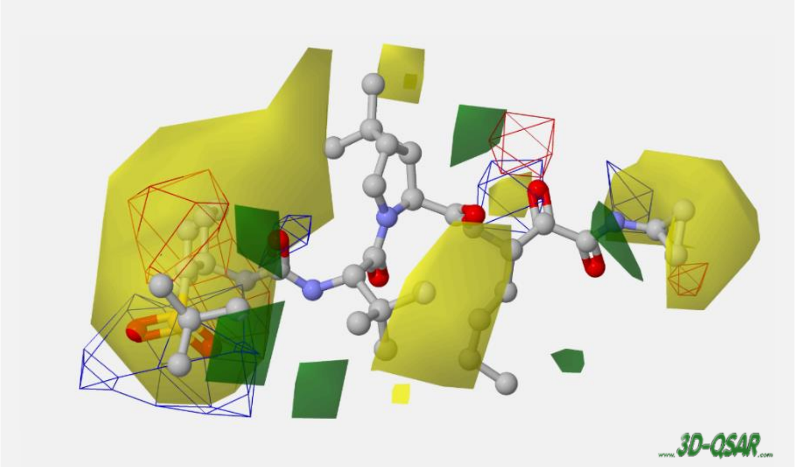	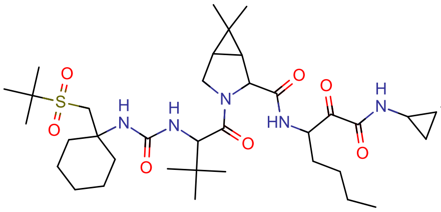	5.1<br>5.73
**20**	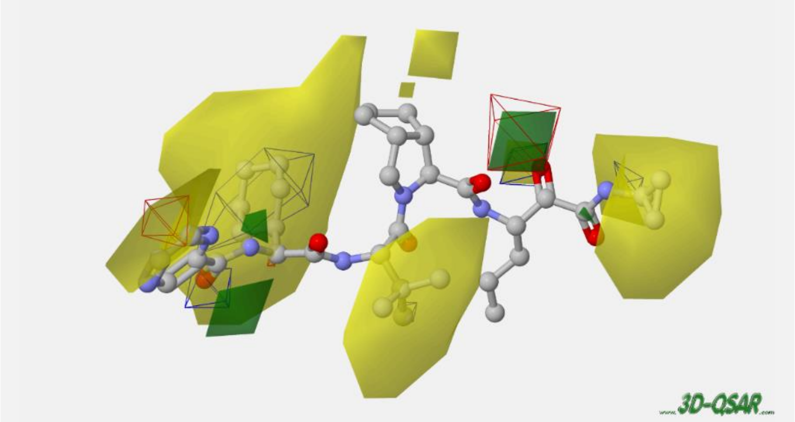	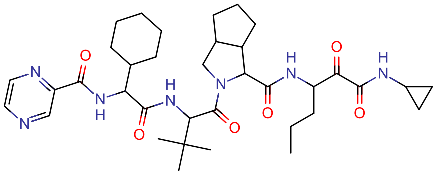	18.0

**21**	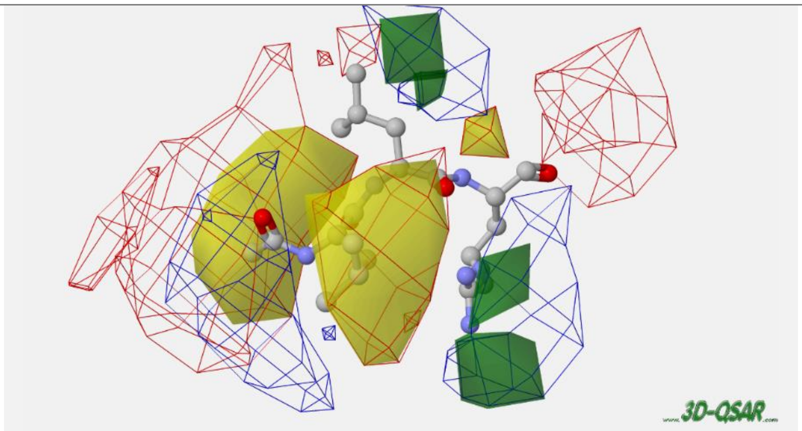	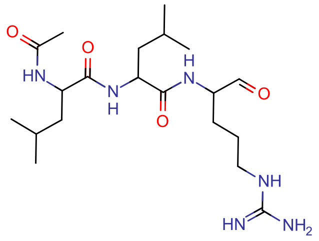	92.0

Figure SI2. Activity contribution (AC) contour plots and 2-D structures of training set molecules **1-21** by LB1 model. Contour levels: 70% (steric: positive green. negative yellow; electrostatic: positive blue. negative red). Hydrogen atoms are omitted for the sake of clarity. Compounds are reported with the same orientation of compound 3 in Figure 3 and 7 (P4 – P1’). The plots were generated directly within the www.3d-qsar.com portal.

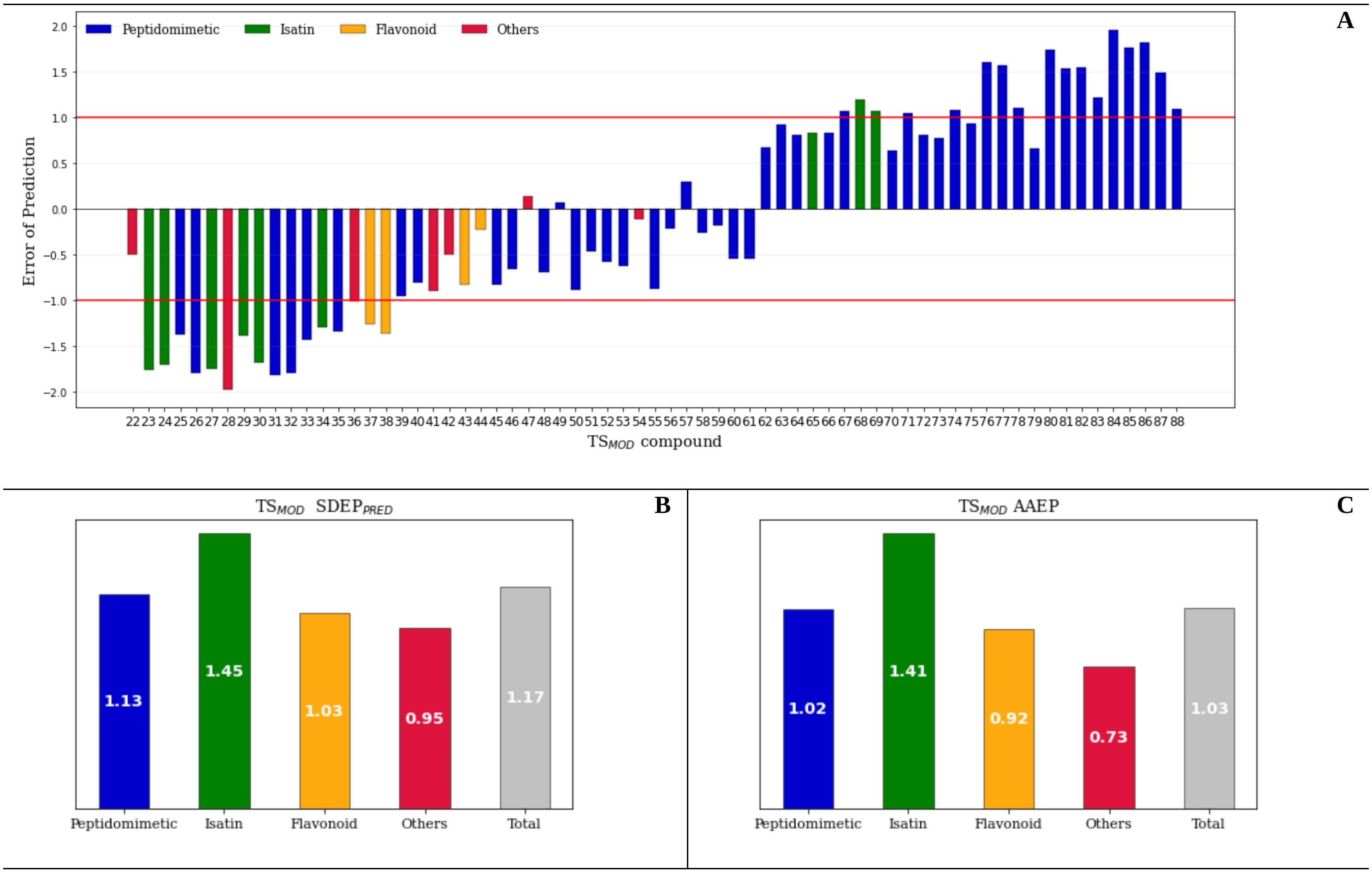

Figure SI3. LB1 model errors of prediction (**A**), standard deviation of prediction error ($SDEP_{pred}$) (**B**) and absolute average error of prediction (AAEP) (**C**) plots of TS_{MOD} test set.

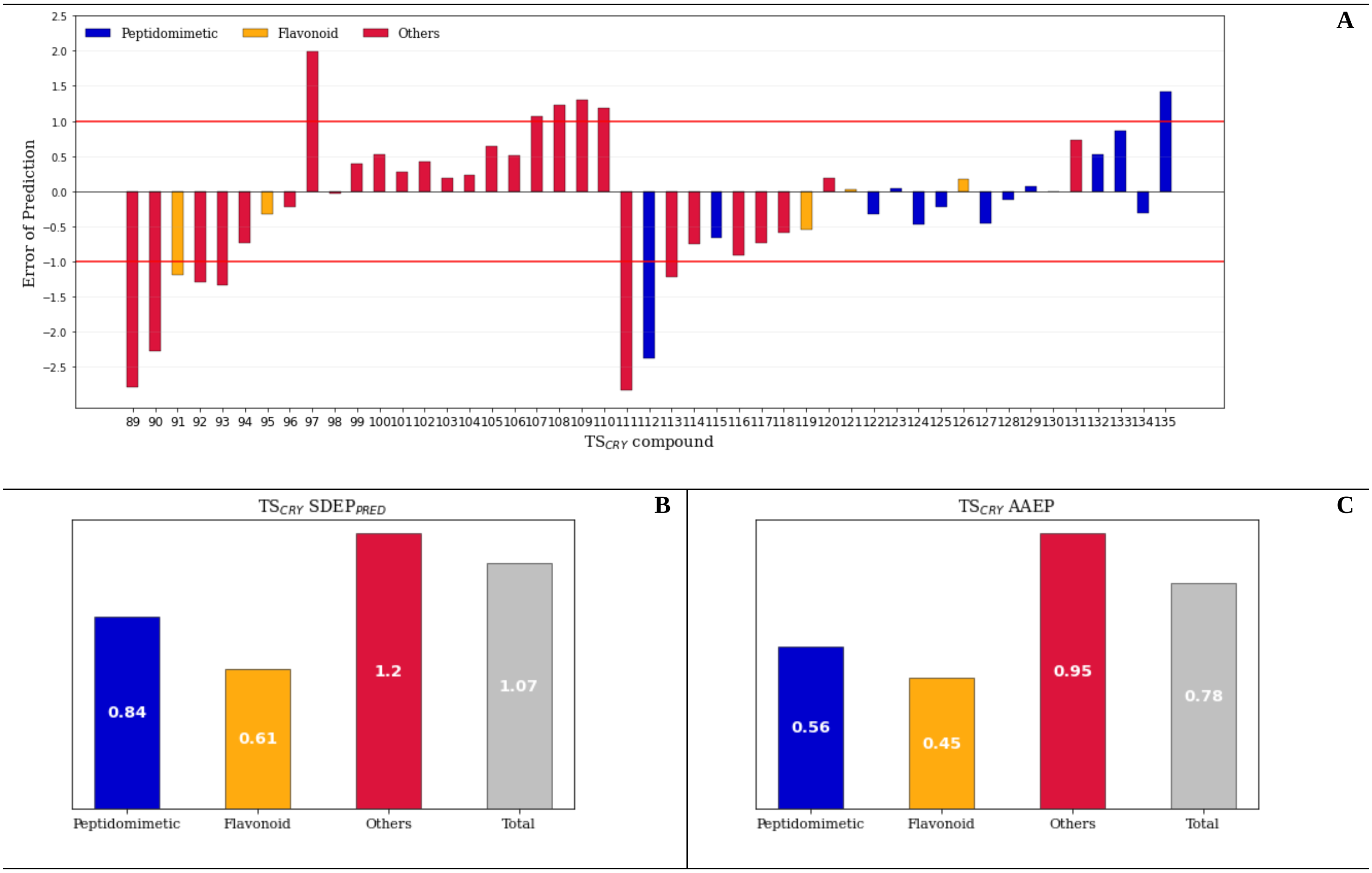

Figure SI4. LB1 model errors of prediction (**A**), standard deviation of prediction error ($SDEP_{pred}$) (**B**) and absolute average error of prediction (AAEP) (**C**) plots of TS_{CRY} test set.

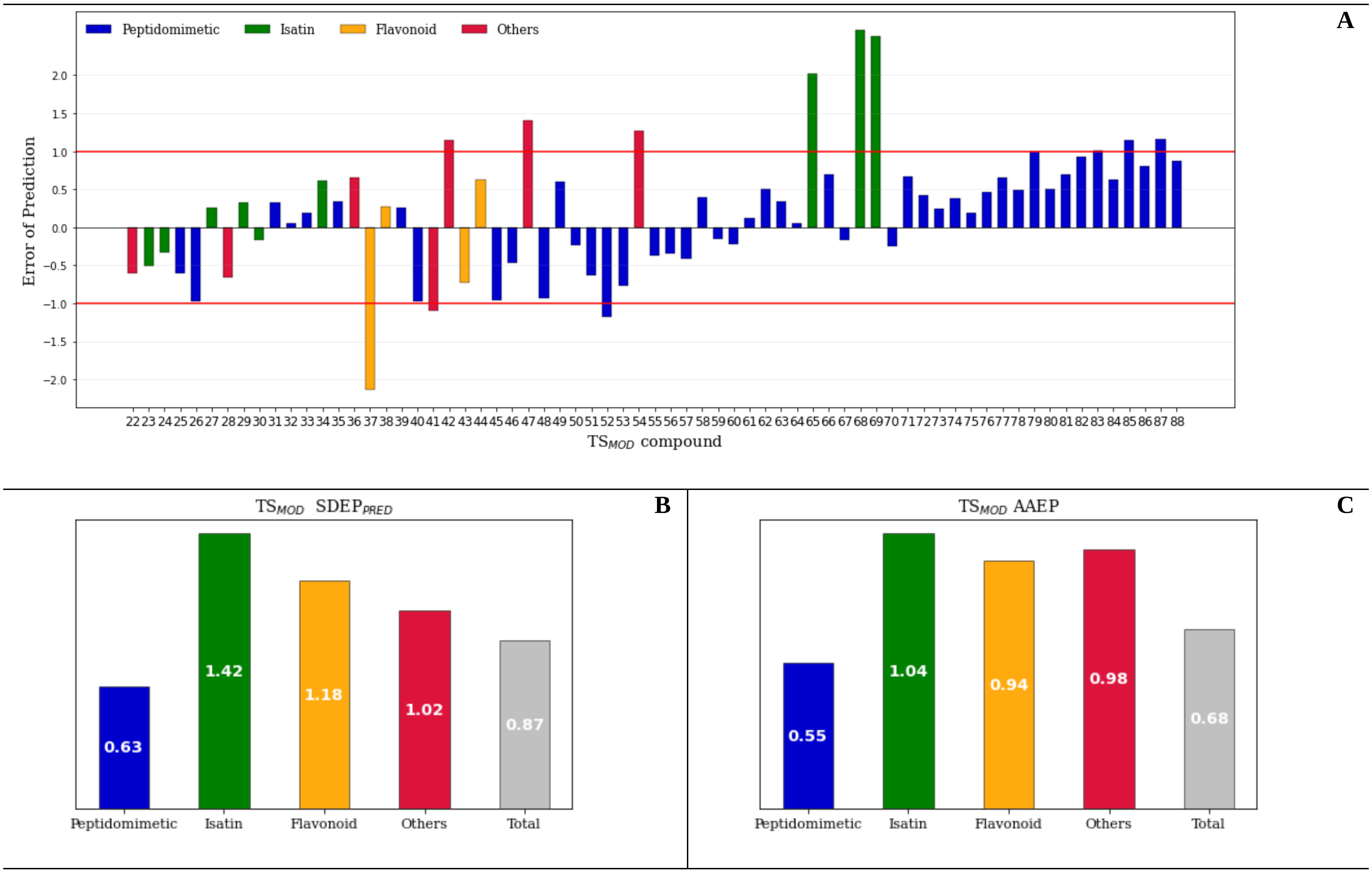

Figure SI5. SB1$_{SAFS}$ model errors of prediction (**A**), standard deviation of prediction error (SDEP$_{pred}$) (**B**) and absolute average error of prediction (AAEP) (**C**) plots of TS$_{MOD}$ test set.

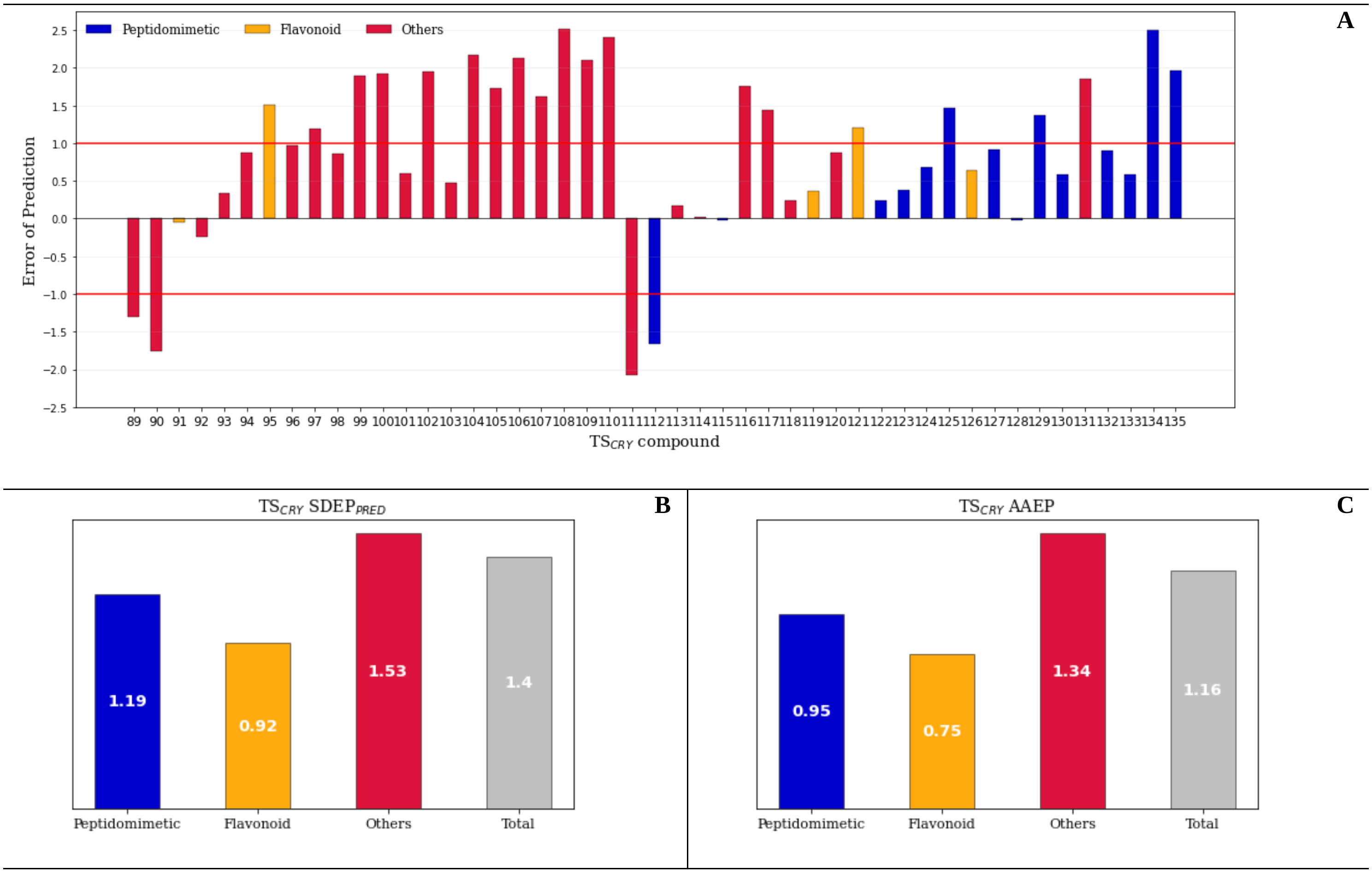

Figure SI6. SB1$_{SAFS}$ model errors of prediction (**A**), standard deviation of prediction error (SDEP$_{pred}$) (**B**) and absolute average error of prediction (AAEP) (**C**) plots of TS$_{CRY}$ test set.

		Experimental Values	
		**Active**	**Non-Active**
**Predicted Values**	**Active**	True Positive (TP)	False Positive (FP)
	**Non-Active**	False Negative (FN)	True Negative (TN)

A

$$Accuracy = \frac{TP + TN}{TP + FP + TN + FN}$$

$$Precision = \frac{TP}{TP + FP}$$

$$Recall = \frac{TP}{TP + FN}$$

B

Figure SI7. Confusion matrix (**A**) and accuracy, precision (positive predicted value), recall (true positive rate) classification metrics (**B**).

References

1. Dai, W., et al., *Structure-based design of antiviral drug candidates targeting the SARS-CoV-2 main protease.* Science, 2020. **368**(6497): p. 1331-1335.
2. Hattori, S.-i., et al., *A small molecule compound with an indole moiety inhibits the main protease of SARS-CoV-2 and blocks virus replication.* Nature Communications, 2021. **12**(1): p. 668.
3. Hoffman, R.L., et al., *Discovery of Ketone-Based Covalent Inhibitors of Coronavirus 3CL Proteases for the Potential Therapeutic Treatment of COVID-19.* J Med Chem, 2020. **63**(21): p. 12725-12747.
4. Jin, Z., et al., *Structure of M(pro) from SARS-CoV-2 and discovery of its inhibitors.* Nature, 2020. **582**(7811): p. 289-293.
5. Kneller, D.W., et al., *Malleability of the SARS-CoV-2 3CL M(pro) Active-Site Cavity Facilitates Binding of Clinical Antivirals.* Structure, 2020. **28**(12): p. 1313-1320 e3.
6. Cannalire, R., et al., *Targeting SARS-CoV-2 Proteases and Polymerase for COVID-19 Treatment: State of the Art and Future Opportunities.* J Med Chem, 2020.
7. Liu, H., et al., *Scutellaria baicalensis extract and baicalein inhibit replication of SARS-CoV-2 and its 3C-like protease in vitro.* J Enzyme Inhib Med Chem, 2021. **36**(1): p. 497-503.
8. Su, H.X., et al., *Anti-SARS-CoV-2 activities in vitro of Shuanghuanglian preparations and bioactive ingredients.* Acta Pharmacol Sin, 2020. **41**(9): p. 1167-1177.
9. Ma, C., et al., *Boceprevir, GC-376, and calpain inhibitors II, XII inhibit SARS-CoV-2 viral replication by targeting the viral main protease.* Cell Res, 2020. **30**(8): p. 678-692.
10. Rathnayake, A.D., et al., *3C-like protease inhibitors block coronavirus replication in vitro and improve survival in MERS-CoV-infected mice.* Sci Transl Med, 2020. **12**(557).
11. Sacco, M.D., et al., *Structure and inhibition of the SARS-CoV-2 main protease reveal strategy for developing dual inhibitors against M(pro) and cathepsin L.* Sci Adv, 2020. **6**(50).
12. Zhang, L., et al., *Crystal structure of SARS-CoV-2 main protease provides a basis for design of improved alpha-ketoamide inhibitors.* Science, 2020. **368**(6489): p. 409-412.
13. Yang, K.S., et al., *A Quick Route to Multiple Highly Potent SARS-CoV-2 Main Protease Inhibitors*.* ChemMedChem, 2020.
14. Fu, L., et al., *Both Boceprevir and GC376 efficaciously inhibit SARS-CoV-2 by targeting its main protease.* Nat Commun, 2020. **11**(1): p. 4417.
15. Vuong, W., et al., *Feline coronavirus drug inhibits the main protease of SARS-CoV-2 and blocks virus replication.* Natue Communications, 2020. **11**(1): p. 4282.
16. Galasiti Kankanamalage, A.C., et al., *Design, Synthesis, and Evaluation of Novel Prodrugs of Transition State Inhibitors of Norovirus 3CL Protease.* J Med Chem, 2017. **60**(14): p. 6239-6248.
17. Cheng, Y. and W.H. Prusoff, *Relationship between the inhibition constant (K1) and the concentration of inhibitor which causes 50 per cent inhibition (I50) of an enzymatic reaction.* Biochem Pharmacol, 1973. **22**(23): p. 3099-108.
18. Li, Z., et al., *Identify potent SARS-CoV-2 main protease inhibitors via accelerated free energy perturbation-based virtual screening of existing drugs.* Proc Natl Acad Sci U S A, 2020. **117**(44): p. 27381-27387.
19. Ma, C. and J. Wang, *Dipyridamole, chloroquine, montelukast sodium, candesartan, oxytetracycline, and atazanavir are not SARS-CoV-2 main protease inhibitors.* Proc Natl Acad Sci U S A, 2021. **118**(8).
20. Li, Z., et al., *Reply to Ma and Wang: Reliability of various in vitro activity assays on SARS-CoV-2 main protease inhibitors.* Proc Natl Acad Sci U S A, 2021. **118**(8).
21. Qiao, J., et al., *SARS-CoV-2 M(pro) inhibitors with antiviral activity in a transgenic mouse model.* Science, 2021. **371**(6536): p. 1374-1378.
22. Bursulaya, B.D., et al., *Comparative study of several algorithms for flexible ligand docking.* Journal of Computer-Aided Molecular Design, 2003. **17**(11): p. 755-763.
23. Tetko, I.V., et al., *Critical assessment of QSAR models of environmental toxicity against Tetrahymena pyriformis: focusing on applicability domain and overfitting by variable selection.* J Chem Inf Model, 2008. **48**(9): p. 1733-46.
24. Tropsha, A. and A. Golbraikh, *Predictive QSAR modeling workflow, model applicability domains, and virtual screening.* Curr Pharm Des, 2007. **13**(34): p. 3494-504.
25. Sahigara, F., et al., *Comparison of different approaches to define the applicability domain of QSAR models.* Molecules, 2012. **17**(5): p. 4791-810.
26. Rogers, D. and M. Hahn, *Extended-connectivity fingerprints.* J Chem Inf Model, 2010. **50**(5): p. 742-54.
27. Liu, P., et al., *Potent inhibitors of SARS-CoV-2 3C-like protease derived from N-substituted isatin compounds.* Eur J Med Chem, 2020. **206**: p. 112702.
28. Su, H., et al., *Identification of pyrogallol as a warhead in design of covalent inhibitors for the SARS-CoV-2 3CL protease.* Nat Commun, 2021. **12**(1): p. 3623.

29. Hattori, S.I., et al., *A small molecule compound with an indole moiety inhibits the main protease of SARS-CoV-2 and blocks virus replication.* Nat Commun, 2021. **12**(1): p. 668.
30. Yang, K.S., et al., *A Quick Route to Multiple Highly Potent SARS-CoV-2 Main Protease Inhibitors*.* ChemMedChem, 2021. **16**(6): p. 942-948.
31. Sutanto, F., et al., *Combining High-Throughput Synthesis and High-Throughput Protein Crystallography for Accelerated Hit Identification.* Angew Chem Int Ed Engl, 2021. **60**(33): p. 18231-18239.
32. Cantrelle, F.X., et al., *NMR Spectroscopy of the Main Protease of SARS-CoV-2 and Fragment-Based Screening Identify Three Protein Hotspots and an Antiviral Fragment.* Angew Chem Int Ed Engl, 2021. **60**(48): p. 25428-25435.
33. Han, S.H., et al., *Structure-Based Optimization of ML300-Derived, Noncovalent Inhibitors Targeting the Severe Acute Respiratory Syndrome Coronavirus 3CL Protease (SARS-CoV-2 3CL(pro)).* J Med Chem, 2021.
34. Zhang, C.H., et al., *Potent Noncovalent Inhibitors of the Main Protease of SARS-CoV-2 from Molecular Sculpting of the Drug Perampanel Guided by Free Energy Perturbation Calculations.* ACS Cent Sci, 2021. **7**(3): p. 467-475.
35. Lockbaum, G.J., et al., *Crystal Structure of SARS-CoV-2 Main Protease in Complex with the Non-Covalent Inhibitor ML188.* Viruses, 2021. **13**(2).
36. Deshmukh, M.G., et al., *Structure-guided design of a perampanel-derived pharmacophore targeting the SARS-CoV-2 main protease.* Structure, 2021. **29**(8): p. 823-833 e5.
37. Vatansever, E.C., et al., *Bepridil is potent against SARS-CoV-2 In Vitro.* bioRxiv, 2020.
38. Redhead, M.A., et al., *Bispecific repurposed medicines targeting the viral and immunological arms of COVID-19.* Sci Rep, 2021. **11**(1): p. 13208.
39. Ghosh, A.K., et al., *Indole Chloropyridinyl Ester-Derived SARS-CoV-2 3CLpro Inhibitors: Enzyme Inhibition, Antiviral Efficacy, Structure-Activity Relationship, and X-ray Structural Studies.* J Med Chem, 2021. **64**(19): p. 14702-14714.
40. Dampalla, C.S., et al., *Structure-Guided Design of Conformationally Constrained Cyclohexane Inhibitors of Severe Acute Respiratory Syndrome Coronavirus-2 3CL Protease.* J Med Chem, 2021. **64**(14): p. 10047-10058.
41. Iketani, S., et al., *Lead compounds for the development of SARS-CoV-2 3CL protease inhibitors.* Nat Commun, 2021. **12**(1): p. 2016.
42. Xia, Z., et al., *Rational Design of Hybrid SARS-CoV-2 Main Protease Inhibitors Guided by the Superimposed Cocrystal Structures with the Peptidomimetic Inhibitors GC-376, Telaprevir, and Boceprevir.* ACS Pharmacol Transl Sci, 2021. **4**(4): p. 1408-1421.
